# Purine Nucleosides Interfere with c-di-AMP Levels and Act as Adjuvants To Re-Sensitize MRSA To β-Lactam Antibiotics

**DOI:** 10.1128/mbio.02478-22

**Published:** 2022-12-12

**Authors:** Aaron C. Nolan, Merve S. Zeden, Igor Kviatkovski, Christopher Campbell, Lucy Urwin, Rebecca M. Corrigan, Angelika Gründling, James P. O’Gara

**Affiliations:** a Microbiology, School of Biological and Chemical Sciences, University of Galway, Ireland; b Section of Molecular Microbiology and Medical Research Council Centre for Molecular Bacteriology and Infection, Imperial College London, London, United Kingdom; c The Florey Institute, School of Bioscience, University of Sheffield, Sheffield, United Kingdom; New York University School of Medicine

**Keywords:** Staphylococcus aureus, MRSA, purine metabolism, antibiotic adjuvant, β-lactam resistance, c-di-AMP, antibiotic resistance

## Abstract

The purine-derived signaling molecules c-di-AMP and (p)ppGpp control *mecA*/PBP2a-mediated β-lactam resistance in methicillin-resistant Staphylococcus aureus (MRSA) raise the possibility that purine availability can control antibiotic susceptibility. Consistent with this, exogenous guanosine and xanthosine, which are fluxed through the GTP branch of purine biosynthesis, were shown to significantly reduce MRSA β-lactam resistance. In contrast, adenosine (fluxed to ATP) significantly increased oxacillin resistance, whereas inosine (which can be fluxed to ATP and GTP via hypoxanthine) only marginally increased oxacillin susceptibility. Furthermore, mutations that interfere with *de novo* purine synthesis (*pur* operon), transport (NupG, PbuG, PbuX) and the salvage pathway (DeoD2, Hpt) increased β-lactam resistance in MRSA strain JE2. Increased resistance of a *nupG* mutant was not significantly reversed by guanosine, indicating that NupG is required for guanosine transport, which is required to reduce β-lactam resistance. Suppressor mutants resistant to oxacillin/guanosine combinations contained several purine salvage pathway mutations, including *nupG* and *hpt*. Guanosine significantly increased cell size and reduced levels of c-di-AMP, while inactivation of GdpP, the c-di-AMP phosphodiesterase negated the impact of guanosine on β-lactam susceptibility. PBP2a expression was unaffected in *nupG* or *deoD2* mutants, suggesting that guanosine-induced β-lactam susceptibility may result from dysfunctional c-di-AMP-dependent osmoregulation. These data reveal the therapeutic potential of purine nucleosides, as β-lactam adjuvants that interfere with the normal activation of c-di-AMP are required for high-level β-lactam resistance in MRSA.

## INTRODUCTION

Staphylococcus aureus is an opportunistic pathogen responsible for localized skin infections and more severe illnesses, such as bacteremia, sepsis, infectious arthritis, pneumonia, endocarditis, urinary tract infections, and toxic shock syndrome (1, 2). While significant advances have been made in our understandings of the metabolism of S. aureus in recent years, we have only just begun to interrogate the role of central metabolic pathways required for bacterial proliferation in the host, and their contribution to antibiotic resistance.

β-lactam antibiotics remain a gold standard for the treatment of S. aureus infections. Resistance of methicillin-resistant S. aureus (MRSA) to β-lactam antibiotics, such as methicillin, that are not cleaved by bacterial β-lactamases is mediated through the expression of the low affinity penicillin binding protein PBP2a, encoded by the *mecA* gene located on the mobile genetic element; the staphylococcal chromosome cassette (SCC*mec*) (3, 4). The PBP2a transpeptidase cross-links peptidoglycan in the presence of β-lactam antibiotics to confer resistance (4–6).

Carriage of SCC*mec* is linked to heterogenous, low level resistance (HeR) to β-lactam antibiotics (4, 7). Deregulation of *mecA* transcription occurs when the MecR sensor-protease is activated by the presence of β-lactams resulting in cleavage of the MecI repressor (8). When exposed to β-lactam antibiotics, high-level, homogeneously resistant (HoR) MRSA mutants carrying accessory mutations outside SCC*mec* can also be selected (9, 10). Commonly, these mutations lead to activation of the stringent response (11, 12), increased cyclic di-AMP (c-di-AMP) levels (13–15), changes in the activity of RNA polymerase (16), as well as the ClpXP chaperone-protease complex (17, 18).

Nucleotide second messengers play an important role in the control of β-lactam resistance. Nucleotides are essential precursors used by all cells for DNA, RNA, and signaling molecule synthesis, while the energy carriers, adenosine-triphosphate (ATP) and guanosine-triphosphate (GTP), are produced from their corresponding nucleotides adenine and guanine (19, 20). Bacteria utilize an array of nucleotide secondary messengers to regulate cellular responses to fluctuations in external stimuli and environmental conditions (21). Bacterial nucleotide messengers are typically produced from the building blocks ATP and GTP (22). Refined signal transduction pathways are necessary for S. aureus to survive and multiply during infection. Signal transduction in S. aureus influences virulence gene expression during infection, stress, and antibiotic survival responses (23, 24).

**FIG 1:**
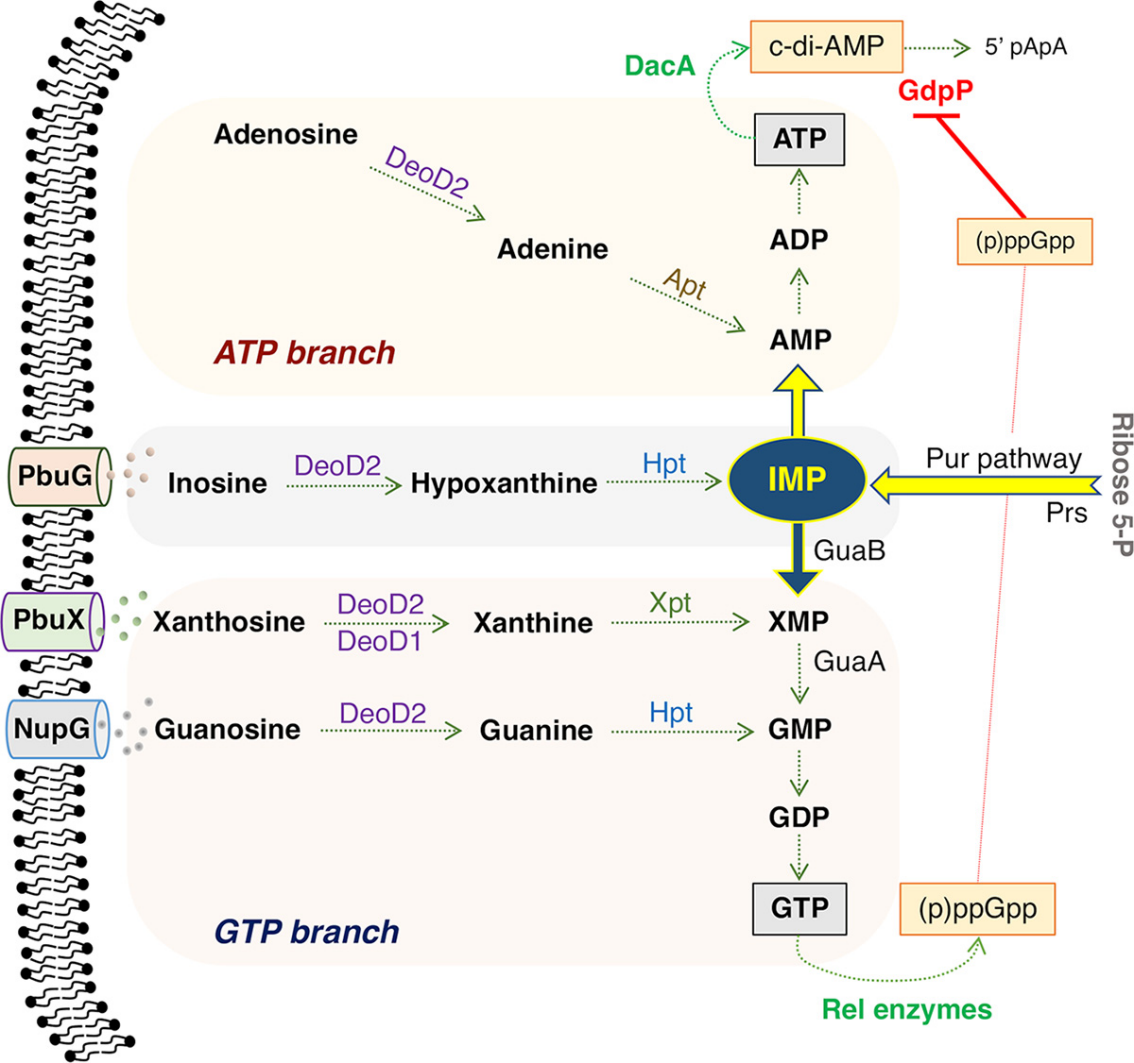
Overview of purine salvage and *de novo* biosynthetic pathways including transporters and enzymes implicated in susceptibility to nucleoside/β-lactam combinations. IMP (IMP) synthesized from ribose 5-P by enzymes encoded by the *pur* operon can be fluxed to ATP and GTP. Nucleotides and nucleosides can be transported into the cell via permeases including NupG, PbuX and PbuG (StgP), where they can be salvaged and returned to the intracellular nucleotide pool via the activity of enzymes including the nucleoside phosphorylases DeoD2 (SAUSA300_2091) and DeoD1 (SAUSA300_0138), hypoxanthine phosphoribosyltransferase Hpt, xanthosine phosphoribosyltransferase Xpt and adenosine phosphoribosyltransferase Apt. Mutations in *hpt*, *prs*, *guaB*, and *guaA* have previously been implicated in the high-level, homogeneous (HoR) β-lactam resistance in MRSA (36). GTP is a substrate for RelA, RelP and RelQ catalyzed synthesis of the stringent response alarmone (p)ppGpp. The cyclic dinucleotide signaling molecule c-di-AMP is synthesized from ATP by DacA, and broken down by GdpP, whose activity is repressed by ppGpp.

S. aureus produces ATP/GTP through a *de novo* 10-step biosynthetic process. The *pur* operon encodes 10 enzymes that first convert ribose-5-phosphate (Ribose-5-P) to IMP; the branchpoint between ATP and GTP synthesis (Fig. 1). IMP can then be converted to AMP or XMP through PurA/PurB or GuaB respectively. AMP is then converted to ADP, ATP, and c-di-AMP. On the alternate branch, XMP is converted to GDP, GTP, and guanosine tetraphosphate/pentaphosphate (ppGpp/(p)ppGpp) which promotes accumulation of c-di-AMP by repressing the activity of the c-di-AMP phosphodiesterase GdpP (25). Alternatively, XMP, GMP, and AMP can be produced from nucleosides/nucleotides through the purine salvage pathway, including transport by the purine transporters PbuX, PbuG, and NupG. There is ongoing interest in purine metabolism due to its multifaceted roles in biofilm formation, virulence, and antibiotic resistance (26–28).

The alarmones ppGpp and pppGpp play a central role in the stringent response, while c-di-AMP contributes to the regulation of cell envelope homeostasis, osmotic regulation, and virulence (29–32). Altering c-di-AMP levels and/or (p)ppGpp levels lead to differences in β-lactam susceptibility of MRSA strains (23, 33). Mutation of the diadenylate cyclase gene *dacA*, leading to reduced c-di-AMP levels, resulted in the conversion of an HoR MRSA to a HeR strain (23). Conversely, inactivating mutations in the c-di-AMP phosphodiesterase gene *gdpP* leading to increased c-di-AMP levels were accompanied by homogeneous resistance to methicillin (13, 14). GdpP activity is repressed by ppGpp, which is synthesized by the Rel, RelP, and RelQ enzymes (33–35). In several studies, it has been reported that activation of the stringent response and constitutive (p)ppGpp production, which will increase c-di-AMP levels (25), are accompanied by homogeneous methicillin resistance (12, 36). The importance of these signaling molecules to bacterial survival under different growth conditions and their absence in humans marks them as attractive antimicrobial drug targets (37).

Further understanding of molecular mechanisms to antibiotic resistance is an important part of efforts to identify new targets with the potential to re-sensitize bacteria to antibiotics, including β-lactams (38). The knowledge gained from understanding such molecular mechanisms could be used to identify compounds that provide synergistic activity with β-lactams, thus providing a potential solution to antibiotic resistance and dwindling antibiotic therapy options (39–41).

In this study, the impact of exogenous purines on growth and susceptibility to β-lactams and other antibiotics is described in several strains of S. aureus and MRSA, and Staphylococcus epidermidis. The contribution of several genes involved in *de novo* purine synthesis, purine transport, purine salvage, and (p)ppGpp/c-di-AMP signaling to nucleoside/β-lactam susceptibility has been elucidated. Our data reveal that re-sensitization of MRSA to β-lactams using guanosine or xanthosine is dependent on several purine transporters and salvage pathway enzymes, and accompanied by increased cell size and reduced levels of c-di-AMP. The therapeutic potential of using purine nucleosides as β-lactam antibiotic adjuvants is discussed.

## RESULTS

### Exogenous purines regulate MRSA growth and susceptibility to β-lactam antibiotics.

The correlation between increased levels of the purine signaling nucleotides (p)ppGpp and c-di-AMP and increased resistance to β-lactam antibiotics raises the possibility that manipulation of purine metabolism (Fig. 1) by controlling the availability of purines in the growth environment can regulate the susceptibility of MRSA to β-lactam antibiotics. Exogenous purines transported into the cell are metabolized via the purine salvage pathway (Fig. 1). Growth of wild-type MRSA strain JE2 at oxacillin (OX) concentrations of 1, 16, 32, or 64 μg/mL was measured in chemically defined media without glucose (CDM) supplemented with both adenine and guanine, without adenine and guanine, with adenine alone or with guanine alone. Weak growth at the lowest OX concentration in CDM with both adenine and guanine was only measured after > 30 h (Fig. S1A). This was significantly improved in CDM lacking both guanine and adenine (Fig. S1B), and growth in OX 1 μg/mL was also measured in CDM with adenine alone (Fig. S1C). Strikingly, in CDM supplemented with guanine alone, no growth of JE2 was measured in OX even after 48 h (Fig. S1D).

10.1128/mbio.02478-22.3 FIG S1 Exogenous guanine and adenine control oxacillin resistance of wild-type JE2 grown in modified chemically defined media. (A) Growth of JE2 for 48 h at 35°C in modified chemically defined media without glucose (CDM) supplemented with 40 mg/l of both adenine and guanine (CDM Ade + Gua) and oxacillin (OX) 1, 16, 32 or 64 μg/mL. (B) Growth of JE2 in CDM No Ade or Gua supplemented with OX 1, 16, 32 or 64 μg/mL. (C) Growth of JE2 in CDM Ade only (no Gua) supplemented with OX 1, 16, 32 or 64 μg/mL. (D) Growth of JE2 in CDM Gua only (no Ade) supplemented with OX 1, 16, 32 or 64 μg/mL. Growth (OD_600_) was measured at 15 min intervals in a Tecan plate reader. Data are the average of 3 independent experiments and error bars represent standard deviation. Download FIG S1, TIF file, 0.8 MB. Copyright © 2022 Nolan et al. 2022 Nolan et al. https://creativecommons.org/licenses/by/4.0/ This content is distributed under the terms of the Creative Commons Attribution 4.0 International license.

**FIG 2:**
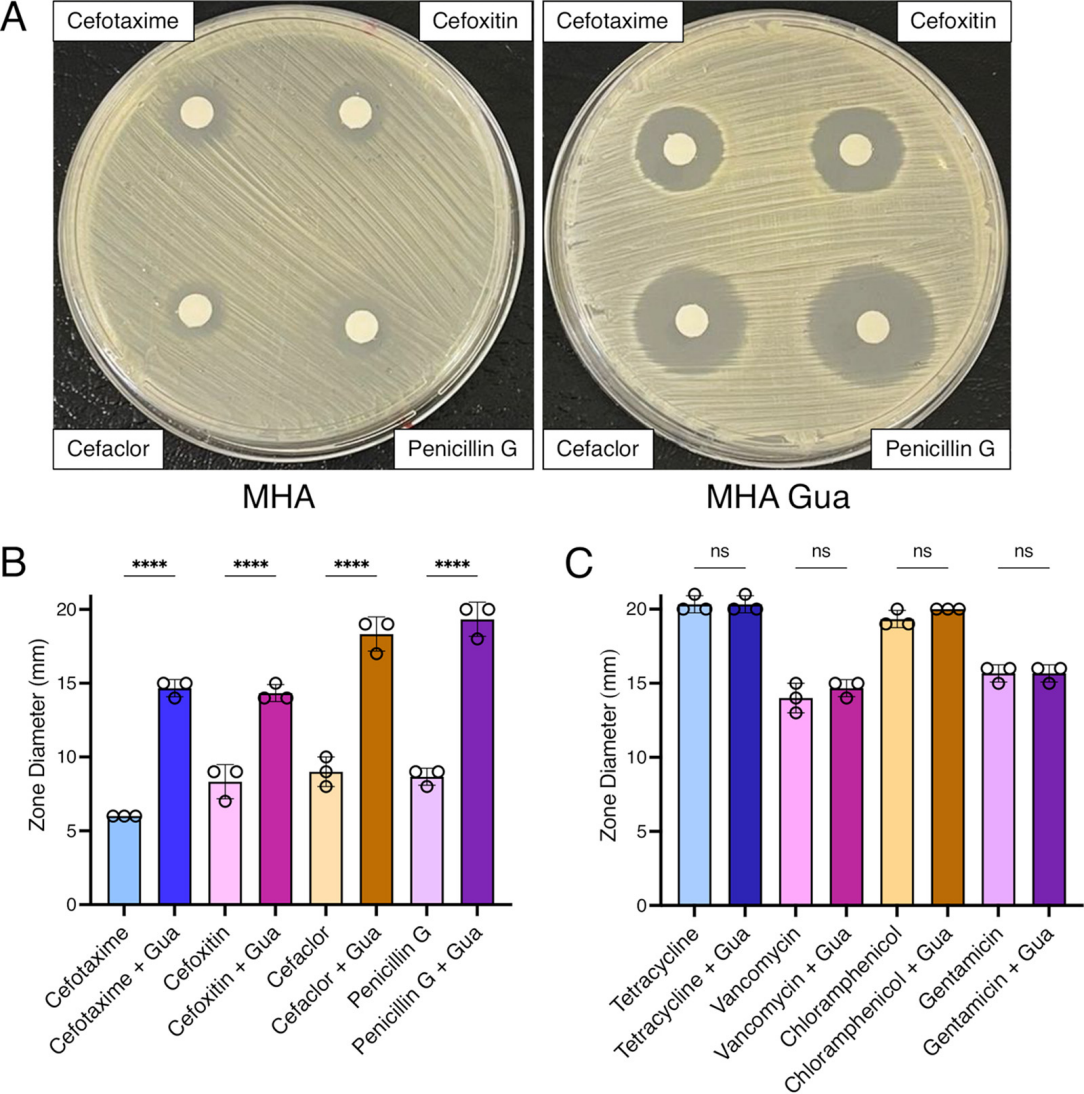
Exogenous guanosine only increases susceptibility of MRSA to β-lactams and not other classes of antibiotic. (A) Susceptibility of wild-type JE2 to the β-lactam antibiotics cefotaxime (top left), cefoxitin (top right), cefaclor (bottom left) and penicillin G (bottom right) determined by disk diffusion assay on Mueller-Hinton 2% NaCl agar (MHA) (left), or MHA supplemented with 0.2 g/l guanosine (MHA Gua) (right). This experiment was repeated three times and a representative image is shown. (B) Susceptibility of wild-type JE2 to the β-lactam antibiotics cefotaxime, cefoxitin, cefaclor and penicillin G determined by disk diffusion assay on MHA or MHA with 0.2 g/l guanosine (Gua). (C) Susceptibility of wild-type JE2 to tetracycline, vancomycin, chloramphenicol or gentamicin determined by disk diffusion assay on MHA, or MHA with 0.2 g/l Gua. Data (zone diameters, mm) are the average of 3 independent experiments and error bars represent standard deviation. Statistical significance was determined by one-way ANOVA. ****, *P* < 0.0001, ns, not significant.

Switching to nucleosides, which are more readily solubilized in water than nucleotides, and Mueller-Hinton 2% NaCl broth (MHB), which is routinely used for antibiotic susceptibility testing, the addition of guanosine or xanthosine rendered JE2 susceptible to OX with the MIC reduced from 64 to ≤ 4 μg/mL (Table 1). In contrast, JE2 was significantly more resistant to OX in MHB supplemented with adenosine (MIC = 256 μg/mL) (Table 1). Interestingly, supplementation of MHB with up to 1.0 g/l inosine, which can be fluxed to both the ATP and GTP branches of purine biosynthesis via hypoxanthine and IMP (Fig. 1), only marginally reduced the JE2 OX MIC to 32 μg/mL. Similarly, OX susceptibility was unchanged in MHB supplemented with both guanosine and adenosine (MIC = 64 μg/mL). Disk diffusion assays on Mueller-Hinton Agar (MHA) showed that exogenous guanosine also increased the susceptibility of JE2 to other β-lactam antibiotics, namely, cefotaxime, cefoxitin, cefaclor, and penicillin G (Fig. 2A and B), but did not show any synergy with tetracycline, vancomycin, chloramphenicol, and gentamicin (Fig. 2C).

**TABLE 1:** Antibacterial activity of oxacillin alone and in combination with guanosine, xanthosine, or adenosine against S. aureus strains, purine metabolism mutants, and S. epidermidis RP62A^*a*^

	Oxacillin/nucleoside combination				
Strain	OX^*b*^	OX/Gua (0.2 g/l)^*c*^	OX/Gua (1 g/l)^*c*^	OX/Xan (1 g/l)^*c*^	OX/Ade (1 g/l)^*c*^
JE2	64	2–4	0.5–1	0.5–1	256
USA300	64	4–8	1–2	1–2	256
MW2	64	32	32	32	ND^*d*^
DAR13	64	8	4–8	8	ND
DAR173	256	16	16	8	ND
DAR169	128	8–16	8–16	16	ND
DAR45	4	2	1	1	ND
DAR22	128	16	8	4–8	ND
S. epidermidis RP62A	128	8	4–8	32	ND
COL	512	512	256	256	ND
BH1CC	256	256	128	128	512
8325–4	0.125	0.06	0.03	0.06	0.25
NE1868 *mecA*	0.25	0.125	0.06	0.125	1
NE529 *purF*	128	8	1	0.5	256
NE522 *purA*	128	2	ND	ND	ND
NE950 *purB*	128	4	ND	ND	ND
NE529 *purC*	128	4	ND	ND	ND
NE581 *purD*	128	4	ND	ND	ND
NE744 *purK*	128	2	ND	ND	ND
NE1785 *purN*	128	4	ND	ND	ND
NE1134 *purS*	128	8	ND	ND	ND
NE353 *purH*	64	8	ND	ND	ND
NE1101 *purM*	128	8	ND	ND	ND
NE1464 *purL*	128	8	ND	ND	ND
NE494 *purQ*	128	8	ND	ND	ND
NE1237 *purR*	32	4–8	1–2	0.5–1	64
NE1419 *nupG*	128	128	128	0.5–1	256
NE283 *pbuG*	128	4	2	0.5	256
NE280 *pbuX*	256	4–8	2	256	256
NE650 *deoD2*	128	128	128	0.5–1	256
NE477 *deoD1*	64	1	0.5–1	0.5–1	256
USA300 R10 *hpt* S_71_C	256	128	128	8	256
USA300 R11 *hpt* T_61_P	256	128	128	16	256
NE1419 pLI50_*nupG*	64	4–8	0.5	0.5	256
NE650 pLI50_*deoD2*	64	4–8	0.5	0.5	256
ANG3165 *rsh*_syn_	128	4–8	1–2	0.5–1	256
ANG1961 Δ*gdpP*::Km	128	128	128	128	128
ANG3664 *dacA* G_206_S	1	0.25	0.125	<0.125	16

a Antibacterial activity, MIC; oxacillin, (OX); guanosine (Gua); xanthosine (Xan); adenosine (Ade).

b MIC values for OX in Mueller-Hinton 2% NaCl broth (MHB); μg/mL.

c MIC values for OX in combination with guanosine (0.2 or 1 g/l), xanthosine (1 g/l) or adenosine (1 g/l) in MHB, μg/mL.

d ND. Not determined.

Checkerboard titration assays further revealed that guanosine increased JE2 susceptibility to OX in a concentration-dependent manner (Fig. S2). In time-kill assays, combinations of guanosine and OX achieved a significant ≥ 2 log^10^ reduction in the number of CFU/mL over 12 h compared to OX alone, and achieved bactericidal activity at OX concentrations > 64 μg/mL (Fig. S3). At lower OX concentrations, recovery of JE2 growth after 12 h, presumably reflected the selection and expansion of suppressors or OX HoR mutants as described previously (13, 42, 43).

10.1128/mbio.02478-22.4 FIG S2 Guanosine increases the oxacillin susceptibility of MRSA strain JE2 in a concentration-dependent manner. Checkerboard titration assays were conducted using guanosine and oxacillin with JE2 grown for 24 h in Mueller-Hinton 2% NaCl broth in 96-well plates. The data shown are the OD_600_ values for each well. The experiments were repeated at least three times and the data from a representative 96-well plate is shown. Purple shaded boxes indicated wells in which significant growth was measured. Download FIG S2, TIF file, 0.8 MB. Copyright © 2022 Nolan et al. 2022 Nolan et al. https://creativecommons.org/licenses/by/4.0/ This content is distributed under the terms of the Creative Commons Attribution 4.0 International license.

10.1128/mbio.02478-22.5 FIG S3 *In vitro* kill curves for wild-type JE2 in MHB, MHB guanosine (Gua, 200 μg/mL), MHB oxacillin (OX, 32 μg/mL, 0.5× MIC; 64 μg/mL, 1.0× MIC; 128 μg/mL, 2.0× MIC) and combinations of Gua and OX. Three-hour cultures were adjusted to 10^6^ CFU/mL in Mueller Hinton 2% NaCl broth (MHB, OD_600_=0.001) or MHB supplemented with Gua and/or OX (Ox). Cultures were incubated at 35°C and the number of CFU/mL enumerated at 0, 2, 4, 6, 8, 12, and 24 h. The data presented are the mean of three independent experiments, and standard error of the mean is shown. Antibiotic synergism was defined as a ≥ 2 log^10^ decrease in the number of CFU/mL in JE2 cell suspensions exposed to OX/Gua combinations compared to Ox alone. Reductions in the number of CFU/mL to ≤10^3^ was defined as bactericidal activity. Download FIG S3, TIF file, 0.5 MB. Copyright © 2022 Nolan et al. 2022 Nolan et al. https://creativecommons.org/licenses/by/4.0/ This content is distributed under the terms of the Creative Commons Attribution 4.0 International license.

This evident synergy between OX and the purine nucleosides guanosine and xanthosine versus the antagonism with adenosine and the relatively neutral effect of inosine implicate purine nucleotide homeostasis in the control of MRSA β-lactam susceptibility.

### Purine nucleoside-regulated β-lactam resistance in other S. aureus strains and S. epidermidis RP62A.

Extending these analyses to other staphylococcal strains revealed that guanosine and xanthosine also reduced the OX MICs of several heterogeneously resistant MRSA strains to varying levels; USA300 (SCC*mec* type IV, CC8), MW2 (SCC*mec* type IV; CC1), DAR13 (SCC*mec* type IV; CC8), DAR173 (SCC*mec* type IV; CC5), DAR169 (SCC*mec* type I; CC8), DAR45 (SCC*mec* type II; CC30), DAR22 (SCC*mec* type III; CC5), and methicillin-resistant S. epidermidis RP62A (Table 1 and Table S1). Guanosine at 1 g/l was only associated with a 50% reduction in the OX MICs of the homogeneously resistant MRSA strains COL (SCC*mec* I, CC8) and BH1CC (SCC*mec* II, CC8) (Table 1), suggesting that genetic changes associated with high-level methicillin resistance can potentially negate the ability of purine nucleosides to reduce β-lactam resistance of MRSA. Interestingly guanosine and xanthosine also significantly reduced the OX MIC for the methicillin susceptible laboratory strain 8325-4 and the Nebraska transposon library (NTML) (44) *mecA* mutant NE1868, while adenosine increased their OX MICs indicating that therapeutic potential of purine nucleosides to control MRSA and MRSE β-lactam susceptibility is both *mecA*-dependent and *mecA*-independent.

10.1128/mbio.02478-22.1 TABLE S1 Bacterial strains and plasmids used in this study. Download Table S1, DOCX file, 0.04 MB. Copyright © 2022 Nolan et al. 2022 Nolan et al. https://creativecommons.org/licenses/by/4.0/ This content is distributed under the terms of the Creative Commons Attribution 4.0 International license.

### Mutations in the *de novo* purine biosynthesis or purine salvage pathways increase resistance to β-lactam antibiotics.

Next, the impacts of mutations in the *de novo* purine synthesis *pur* operon and purine salvage pathways (Fig. 1) on β-lactam resistance were investigated. NTML mutations in all tested *pur* operon genes (except *purH*) increased OX resistance, whereas mutation of the *purR* repressor gene was accompanied by increased OX susceptibility (Table 1). OX resistance was also increased in mutants of the putative transporters *nupG* (NE1419, guanine/guanosine), *pbuG/stgP* (NE283, guanine/hypoxanthine), *pbuX* (NE280, xanthine/xanthosine), and a predicted purine nucleoside phosphorylase *deoD2* (NE650) (Table 1). Consistent with the predicted roles of these nucleoside transporters, xanthosine reduced the oxacillin MIC of the *nupG* mutant and guanosine reduced OX resistance of the *pbuX* mutant (Table 1). These data suggest that NupG is the major guanosine transporter and PbuX is the main xanthosine transporter under the conditions tested. Both guanosine and xanthosine reduced OX resistance of the *pbuG* mutant (Table 1), which was previously shown to transport guanine (45). The very subtle effect of inosine on the OX MIC precluded investigation of the possible roles of NupG, PbuG, and PbuX in inosine transport using susceptibility testing. Interestingly, xanthosine, but not guanosine, significantly reduced the OX MIC of the *deoD2* mutant (Table 1), indicating that xanthosine can be phosphorylated by a different enzyme during purine salvage. The most likely candidate is a second putative nucleoside phosphorylase DeoD1 (NE477), which shares 67.4% identity with DeoD2 (Fig. S4A). Mutation of *deoD1* alone did not impact oxacillin susceptibility, and both guanosine and xanthosine reduced the OX MIC of this mutant (Table 1), suggesting that DeoD1 and DeoD2 may be able to substitute for each other in the phosphorylation of xanthosine under specific growth conditions. Finally, in keeping with the prediction that guanine and xanthine are processed by different phosphoribosyltransferase enzymes (Hpt and Xpt, respectively, Fig. 1), xanthosine but not guanosine also reduced the OX MIC by 4–5 fold in the *hpt* mutants R10 and R11 carrying S_71_C and T_61_P substitutions, respectively (45) (Table 1).

10.1128/mbio.02478-22.6 FIG S4 Chromosomal organization of *deoD1*, *nupG, and deoD2* and Western blot analysis of PBP2a in the NE1419 (*nupG*) and NE650 (*deoD2*) mutants. (A) Location of the transposon insertion in NE1419 (*nupG*::Em^r^) and the chromosomal locus amplified and cloned into pLI50_*nupG* for complementation experiments. (B) Location of the transposon insertion in NE650 (*deoD2*::Em^r^) and the chromosomal locus amplified and cloned into pLI50_*deoD2* for complementation experiments. (C) Location of the transposon insertion in NE477 (*deoD1*::Em^r^). (D) Western blot of PBP2a protein in wild-type JE2, NE1419 (*nupG*), NE650 (*deoD2*), NE1419 pLI50_*nupG*, NE650 pLI50_*deoD2*, MSSA strain 8325-4 (negative control), HoR MRSA strain BH1CC (positive control) and JE2 (2^nd^ well). The strains were grown overnight in MHB 2% NaCl supplemented with 5 μg/mL oxacillin (OX), except for BHICC which was grown in MHB NaCl supplemented with 32 μg/mL OX and 8325-4 which was grown in MHB NaCl with no OX. For each sample, 6 μg total protein was run on a 7.5% Tris-Glycine gel, transferred to a PVDF membrane and probed with anti-PBP2a (1:1000), followed by HRP-conjugated protein G (1:2000) and colorimetric detection with Opti-4CN Substrate kit. Three independent experiments were performed, and a representative image is shown. Download FIG S4, TIF file, 1.0 MB. Copyright © 2022 Nolan et al. 2022 Nolan et al. https://creativecommons.org/licenses/by/4.0/ This content is distributed under the terms of the Creative Commons Attribution 4.0 International license.

To verify that the *nupG* and *deoD2* mutations were responsible for increased OX resistance in NE1419 and NE650, the *nupG*::Erm^r^ and *deoD2*::Erm^r^ alleles were transduced using phage 80α into wild-type JE2 and MW2 (Table S1). The OX MICs of the resulting transductants were the same as NE1419 and NE650, i.e., 128 μg/mL. The NE1419 and NE650 mutants were also complemented by the respective wild-type genes cloned into pLI50 (Fig. S4B and C, and Table 1), restoring OX sensitivity and confirming the specific role of each in the resistance phenotype. Using Western blot analysis, no differences were detected in PBP2a expression levels in these mutants and their complemented derivatives compared to wild-type JE2 (Fig. S4D). Collectively, these data are consistent with the role of purine biosynthesis and salvage in the regulation of MRSA β-lactam resistance via a mechanism that is independent of PBP2a.

### Exogenous nucleosides control MRSA growth, cell size, and β-lactam susceptibility in a NupG-dependent manner.

**FIG 3:**
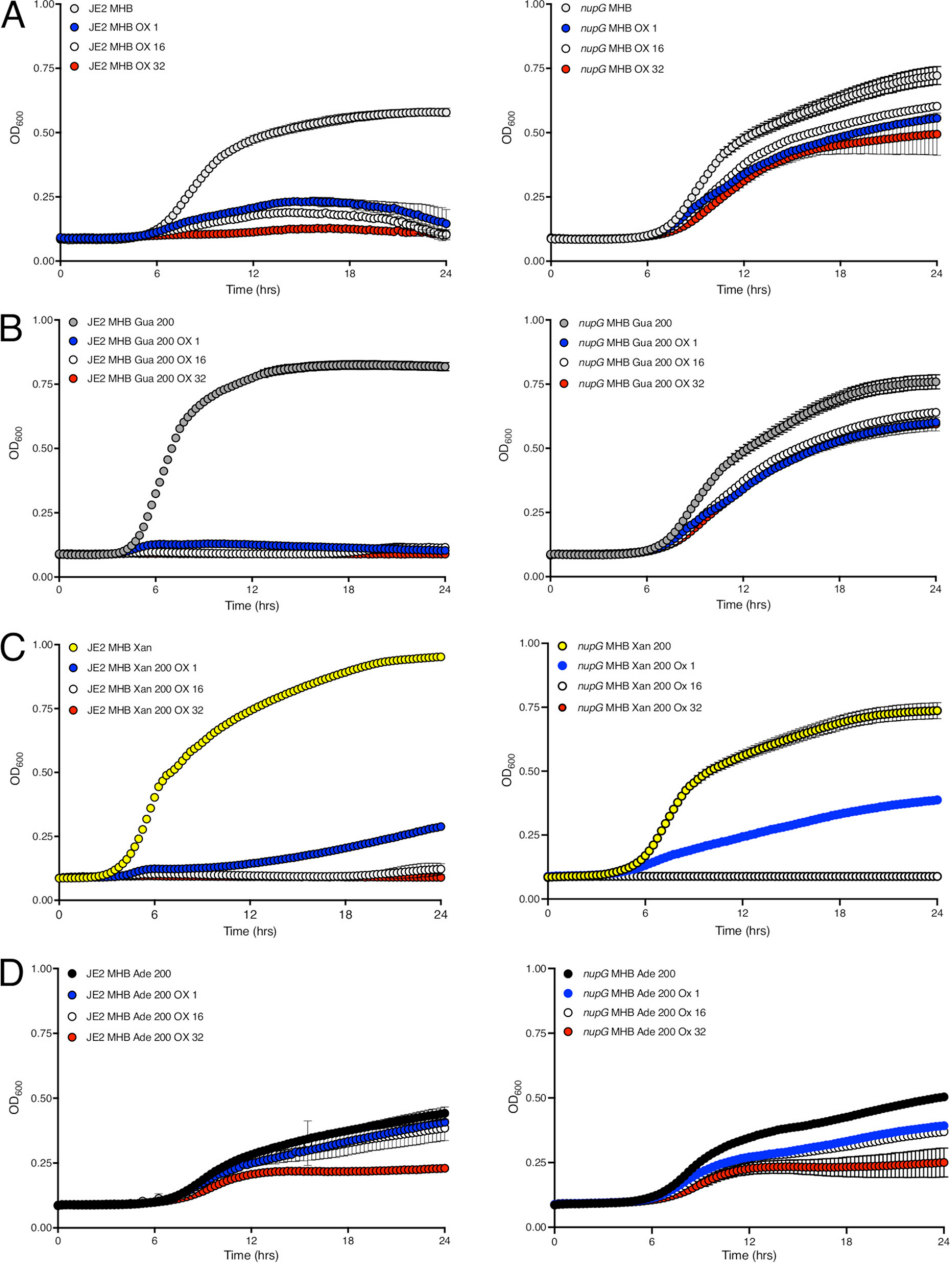
The *nupG* mutant NE1419 is more resistant to oxacillin and is not susceptible to oxacillin-guanosine combinations. (A) Growth of wild-type and NE1419 (*nupG*) for 24 h at 35°C in Mueller-Hinton 2% NaCl broth (MHB) and MHB supplemented with oxacillin (OX) 1, 16, or 32 μg/mL. (B) Growth of wild-type and NE1419 (*nupG*) in MHB Gua 0.2g/l and MHB Gua 0.2 g/l supplemented with OX 1, 16 or 32 μg/mL. (C) Growth of wild-type and NE1419 (*nupG*) in MHB Xan 0.2 g/l and MHB Xan 0.2 g/l supplemented with OX 1, 16 or 32 μg/mL. (D) Growth of wild-type and NE1419 (*nupG*) in MHB Ade 0.2 g/l and MHB Ade 0.2 g/l supplemented with OX 1, 16 or 32 μg/mL. Growth (OD_600_) was measured at 15 min intervals in a Tecan plate reader. Data are the average of 3 independent experiments and error bars represent standard deviation.

Given their role as potential carbon sources, the impact of exogenous nucleosides on the growth of MRSA, which may in turn influence antibiotic resistance, was investigated. Weak JE2 growth in OX 1, 16, or 32 μg/mL (Fig. 3A) was completely inhibited by 0.2 g/l guanosine (Fig. 3B). In contrast, the increased OX resistance of the *nupG* mutant was not significantly reversed by guanosine (Fig. 3A and B), indicating again that NupG is the main guanosine transporter in S. aureus under the growth conditions tested. JE2 growth in OX was also inhibited by 0.2 g/l xanthosine (Fig. 3C), but was unaffected in MHB 0.2 g/l adenosine (Fig. 3D). Consistent with the likely role of PbuX in xanthosine transport, growth of the *nupG* mutant in OX was significantly inhibited by xanthosine (Fig. 3C), and, like the wild-type, was largely unaffected by adenosine (Fig. 3D).

**FIG 4:**
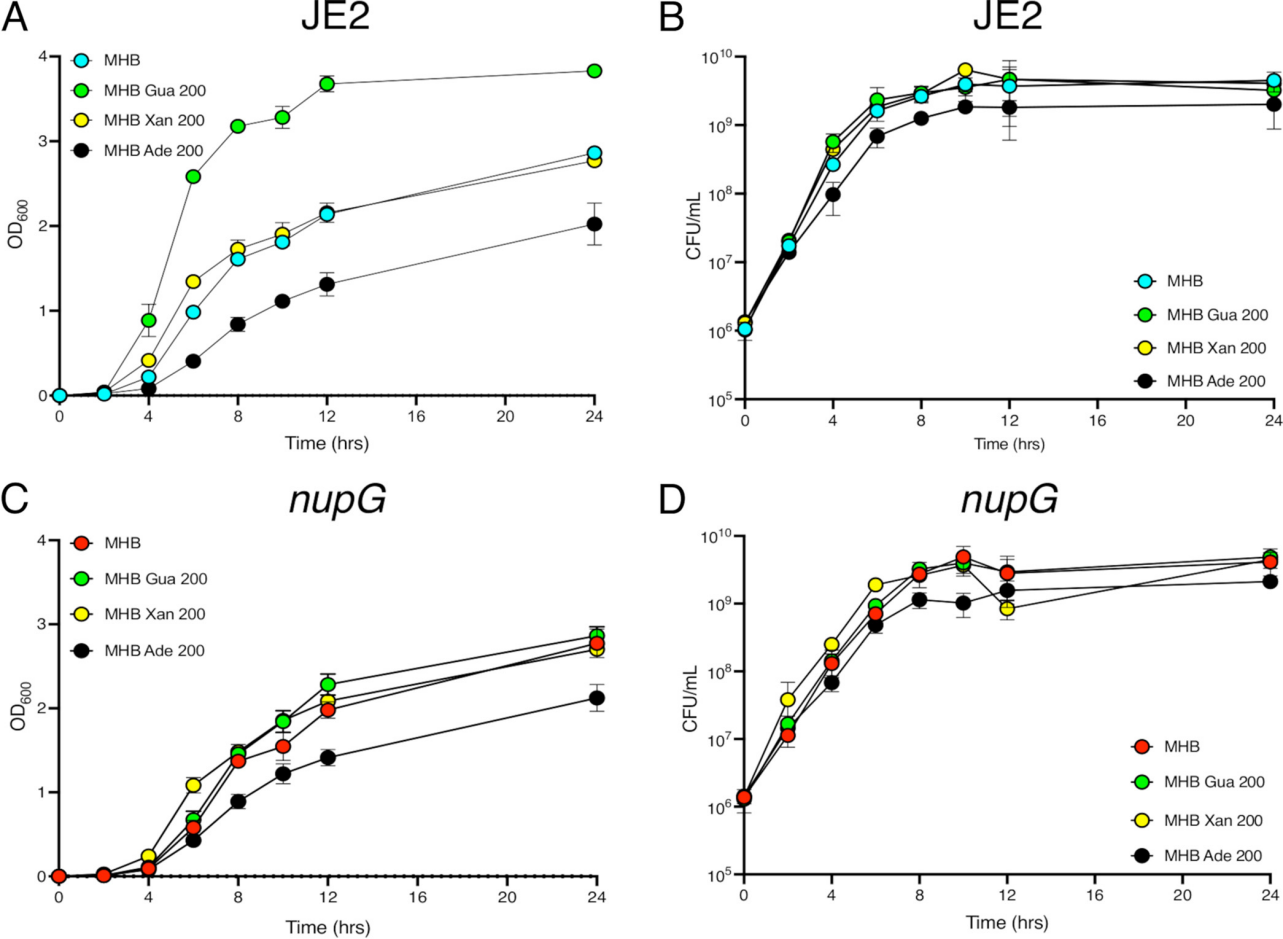
Exogenous guanosine, xanthosine and adenosine impact growth and oxacillin susceptibility in a NupG-dependent manner. Growth of wild-type and NE1419 (*nupG*) at 35°C in Mueller-Hinton 2% NaCl broth (MHB) or MHB supplemented with 0.2 g/l guanosine (Gua), Xanthosine (Xan) or adenosine (Ade) was compared by measuring cell density (OD_600_) (A and C) or enumerating CFU (CFU/mL) (B and D) after 0, 2, 4, 6, 8, 12, and 24 h. Both strains were grown in 25 mL culture volumes in 250 mL flasks (1:10 ratio). Data are the average of 3 independent experiments and error bars represent standard deviation.

Strikingly, guanosine significantly increased the optical density (OD_600_) of JE2 cultures, whereas adenosine negatively impacted growth and xanthosine had no effect (Fig. 4A). However, enumeration of JE2 CFU revealed no significant differences between MHB or MHB supplemented with guanosine, xanthosine, or adenosine (Fig. 4B). For the *nupG* mutant, exogenous guanosine or xanthosine did not significantly affect growth, but cell density (OD_600_) was significantly reduced by adenosine (Fig. 4C). CFU numbers for *nupG* cultures were unaffected by any nucleoside (Fig. 4D). Given that all 3 of these ribose-containing nucleosides have the potential to serve as carbon sources, their different effects on cell density indicates that their impact on β-lactam susceptibility was not a nutritional effect.

**FIG 5:**
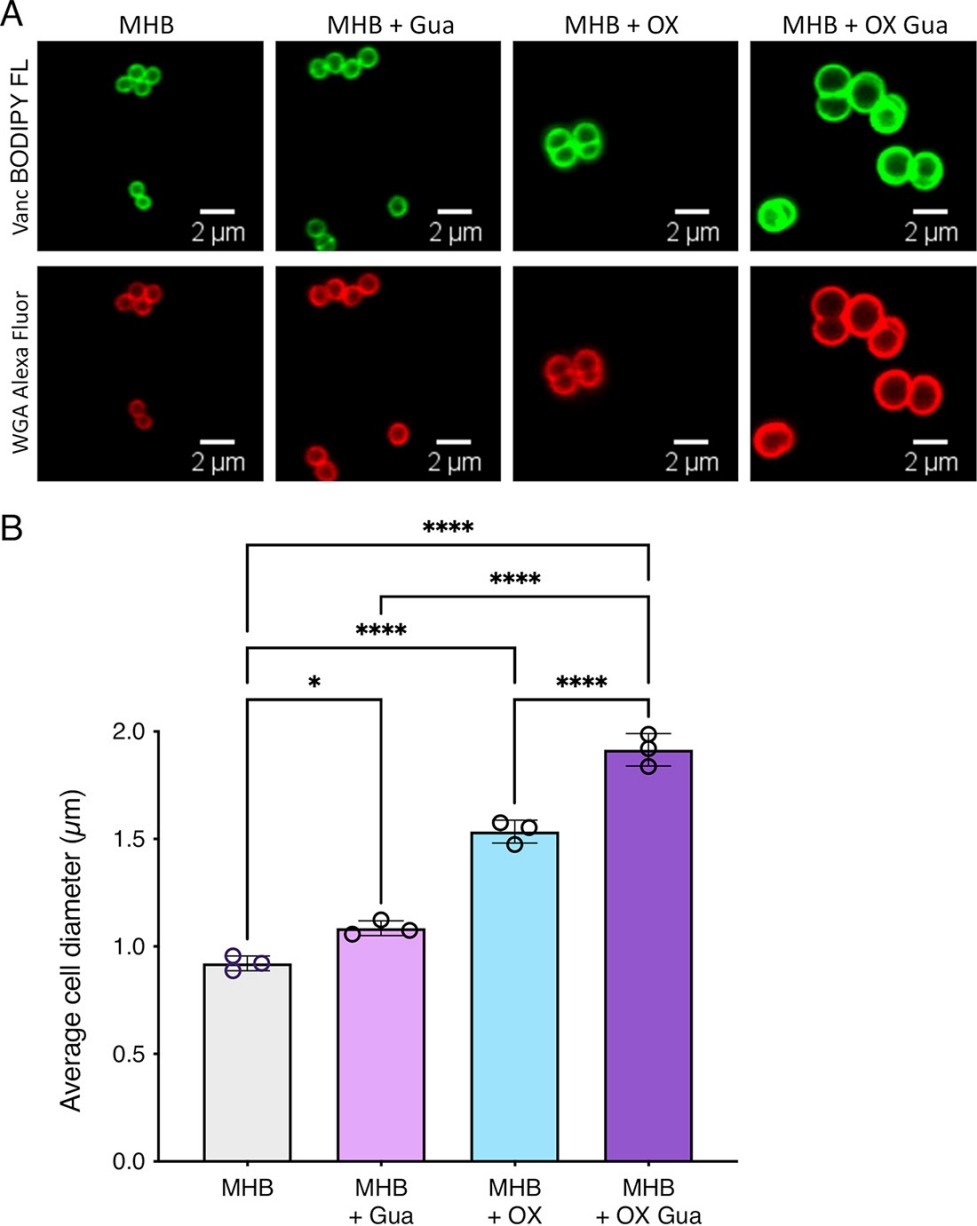
MRSA cell size is dramatically increased by growth in exogenous guanosine and oxacillin. (A) Representative microscopic images of JE2 cells grown in MHB NaCl or MHB NaCl supplemented with guanosine (Gua, 0.2 g/l), oxacillin (OX,1 μg/mL) or a combination of Gua and OX labeled with vancomycin-BODIPY FL (green, top panel) or WGA Alexa Fluor 594 (red, bottom panel). (B) Average diameter of JE2 cells grown in MHB NaCl or MHB NaCl supplemented with Gua (0.2 g/l), OX (1 μg/mL) or a combination of Gua and OX. Images of cells from 3 biological replicates were acquired using Fv3000 confocal microscope and software, 40 cells measured per biological replicate (120 cells in total per condition), and the average and standard deviations for the 3 biological replicates were plotted using GraphPad Prism V9. Asterisks indicate statistically significant difference according to one-way ANOVA followed by Tukey’s multiple comparison *post hoc* test (*, *P* < 0.05; **, *P* < 0.01; ***, *P* < 0.001; ****, *P* < 0.0001). Error bars indicate standard deviation.

Exposure to β-lactam antibiotics has previously been shown to increase S. aureus cell size and lead to cell division defects (46–49). To investigate the impact of exogenous guanosine on morphology and cell size, which can impact growth culture optical density, confocal microscopy experiments were performed on cells collected from JE2 cultures grown in MHB NaCl, MHB NaCl OX, MHB NaCl Gua, and MHB NaCl OX/Gua and stained with Vancomycin-BODIPY or WGA Alexa Fluor 594 (Fig. 5A and B). The diameter of cells grown in guanosine was slightly, yet significantly, increased compared to cells grown in MHB NaCl only. Cell size was further increased in cells grown in oxacillin, with the largest cell size measured in the OX/Gua combination (Fig. 5A and B). In contrast, exogenous adenosine did not affect cell size (Fig. S6A), while the average diameter of cells exposed to an oxacillin/adenosine combination was decreased (Fig. S6B).

10.1128/mbio.02478-22.8 FIG S6 Impact of exogenous guanosine or mutations in *nupG* and *deoD* on GTP and ppGpp levels. (A) GTPase-Glo bioluminescence assay of GTP levels in JE2, *nupG* and *deoD2* grown in MHB NaCl supplemented with guanosine (Gua, 0.2 g/l) and/or oxacillin (OX, 1 μg/mL). (B) Radiochemical thin layer chromatographic analysis of ppGpp levels in JE2, *nupG* and *deoD2* grown in MHB NaCl with Gua 0.2 g/l and/or OX 1 μg/mL. Data are the average of 3 independent experiments and error bars indicate standard deviation. Asterisks indicate statistically significant differences according to one-way ANOVA followed by Tukey’s multiple comparison post-hoc test (*, *P* < 0.05; ***, *P* < 0.001). Download FIG S6, TIF file, 0.7 MB. Copyright © 2022 Nolan et al. 2022 Nolan et al. https://creativecommons.org/licenses/by/4.0/ This content is distributed under the terms of the Creative Commons Attribution 4.0 International license.

Increased cell size may explain, at least in part, why exogenous guanosine increased the optical density (OD_600_) of JE2 cultures, independent of changes in the number of CFU. Furthermore, the dramatic effect of the OX/Gua combination on both increased β-lactam susceptibility and cell size raised the possibility that these phenotypes are associated with altered regulation of c-di-AMP, which as noted earlier is known to control both of these phenotypes (29–32).

### Suppressor mutations in purine metabolism, transcriptional and translational regulation, and ClpX overcome MRSA susceptibility to oxacillin/purine combinations.

Repetitive passaging experiments in MHB 2% NaCl supplemented with OX (32 μg/mL) and guanosine (0.2 g/l) were used to isolate suppressor mutants resistant to the OX/Gua combination. For control purposes, HoR mutants isolated from JE2 grown in medium supplemented with OX alone (32 μg/mL) were also isolated. In total, 16 OX/Gua suppressor mutants and 4 HoR mutants that exhibited stable resistance to oxacillin were characterized and sequenced (Table 2). All 16 OX/Gua suppressor mutants exhibited higher MICs to OX, OX/Gua, and OX/Xan combinations (Table 2). Several mutations directly impacted purine biosynthetic and salvage pathways supporting the hypothesis that purine homeostasis controls β-lactam resistance. The identification of a W_29_Stop suppressor mutation in *nupG* (GS28) is consistent with the phenotype of the *nupG* transposon mutant (NE1419) described above. A *hpt* L_113_Stop mutation was identified in suppressors GS8 and GS25 (Table 2) (Fig. 1). Consistent with the increased β-lactam resistance of GS8 and GS25, the R10 and R11 *hpt* mutants (45) were also found to have an increased oxacillin MIC = 256 μg/mL (Table 1). However, in contrast the R10 and R11 *hpt* mutants (45) (Table 1), the oxacillin MIC of the GS8 and GS25 *hpt* null mutants was not substantially reduced by xanthosine (Table 2), presumably due to the loss of Hpt activity in G8 and GS25 versus altered Hpt activity of R10 and R11. Suppressor mutants GS15, GS16, GS22, and GS23 carry G_35_D mutations in ribose-phosphate pyrophosphokinase (Prs), which is responsible for the synthesis of phosphoribosyl diphosphate (PRPP), the substrate for the *pur* operon-encoded enzymes. Mutations that may phenocopy increased activity of the stringent response required for expression of high-level β-lactam resistance include RpoB mutations (T_480_K in GS4, GS29 and T_622_I in GS10, GS17 and GS28) that lead to increased β-lactam resistance, increased doubling times and thickened cell walls similar to increased Rel activity (36, 50, 51), as well as mutations that potentially impact translation (tRNA-Met in GS3, GS6, GS21, tRNA-Leu in GS10 and tRNA-Ala in GS21, GS27). GS27 has a G_278_Stop substitution in ClpX, which is known to control β-lactam resistance (17). GS28 has a A_344_T substitution in FemA previously implicated in resistance (52), as well as additional mutations in *rpoB* and *nupG*. Similarly, the PdhB G_65_V mutations in GS16 and GS23 occur in conjunction with Prs G_35_D substitutions and may further enhance growth and resistance in media supplemented with OX/Gua. Mutations in *pdhB* were previously shown to confer a growth advantage under osmotic stress (53).

**TABLE 2:** Antibacterial activity of oxacillin alone and in combination with guanosine or xanthosine, against wild-type MRSA and selected OX/Gua and OX homogenously resistant suppressor mutants^*a*^

	Oxacillin/nucleoside combination			
Strain	OX^*b*^	OX/Gua (1 g/l)^*c*^	OX/Xan (1 g/l)^*c*^	Mutation(s)
Wild-type				
JE2	64	0.5–1	0.5–1	– ^*d*^
OX/Gua suppressors				
GS1	256	256	128	ClpX, G_278_Stop
GS3	256	128	128	tRNA–Met (RS09985)
GS4	256	256	128	RpoB, T_480_K
GS6	256	256	128	tRNA–Met (RS09985)
GS8	256	256	128	Hpt, L_113_Stop
GS10	256	256	128	RpoB, T_622_I; tRNA–Leu (RS10005)
GS15	256	256	128	Prs, G_35_D
GS16	256	256	128	Prs, G_35_D; PdhB G_65_V
GS17	256	128	128	RpoB, T_622_I
GS21	256	128	128	tRNA–Ala (RS02670); tRNA–Met (RS09985)
GS22	256	256	128	Prs, G_35_D; RS06410, G_108_Stop
GS23	256	256	128	Prs, G_35_D; PdhB G_65_V
GS25	256	256	256	Hpt, L_113_Stop
GS27	256	128	128	ClpX, G_278_Stop; tRNA–Ala (RS02670)
GS28	256	256	128	RpoB, T_622_I; NupG, W_29_Stop; FemA, A_344_T
GS29	256	128	128	RpoB, T_480_K; Intergenic
OX suppressors (HoRs)			
OS14	128	256	256	ClpX, G_98_Stop
OS18	128	64	32	RsbW, P_101_H
OS26	128	4	4	PBP2, F_44_S
OS30	128	64	32	RsbW, P_101_H

a Antibacterial activity, MIC; oxacillin (OX); guanosine (Gua); xanthosine (Xan); homogenously resistant, HoR.

b MICs for OX; μg/mL.

c MICs for OX in combination with 1g/l guanosine or xanthosine; μg/mL.

d A HemE L_111_F mutation was identified in JE2 and all of the GS and OS suppressors sequenced in this experiment.

The 4 Ox HoRs isolated for control purposes displayed varying MICs for OX/Gua and OX/Xan, although all were more resistant to the combinations than the wild-type JE2 strain (Table 2). OS14, which has ClpX G_98_Stop mutation was highly resistant to the OX/Gua combination (MIC = 256 μg/mL), and even more resistant to the OX/Xan combination (MIC = 256 μg/mL) than the GS1 and GS27 suppressor mutants with ClpX G_278_Stop mutations (MIC = 128 μg/mL). OS18 and OS30 were found to have P_101_H substitutions in RsbW, the anti-sigma factor that controls the activity of the alternative sigma factor SigB, indicating that, consistent with a previous report (54), an altered σ^B^-mediated stress response can increase β-lactam resistance and partially overcome susceptibility to the OX/Gua and OX/Xan combinations. Finally, a PBP2 F_44_S substitution in OS26 was associated with a modest increase in resistance to oxacillin, and the OX/Gua and OX/Xan combinations (Table 2). Overall, these data reveal that aside from suppressors in the purine salvage pathway, mutations associated with the HoR phenotype can also confer resistance to OX/nucleoside combinations.

### Guanosine-oxacillin combination treatment is associated with a *nupG* and *deoD2*-dependent reduction in c-di-AMP levels.

The ATP and GTP-derived stringent response alarmone (p)ppGpp and nucleotide second messenger c-di-AMP regulate β-lactam resistance in MRSA (14, 23). Comparison of GTP levels using a GTPase-Glo bioluminescence assay surprisingly revealed no significant differences between JE2, *nupG*, and *deoD2* grown in MHB NaCl supplemented with guanosine (0.2 g/l) and/or oxacillin (1 or 32 μg/mL) (Fig. S6A). Using the same strains and growth conditions, radiochemical thin-layer chromatography (TLC) assays also to our surprise revealed no significant differences in (p)ppGpp levels, except in JE2 grown in MHB NaCl supplemented with guanosine (Fig. S6B). The absence of significant changes in the stringent response alarmone in the *nupG* or *deoD2* mutants or during growth in OX/Gua combinations revealed that guanosine/xanthosine-mediated increase in oxacillin susceptibility is independent of (p)ppGpp signaling. Furthermore, the oxacillin MIC of a *rsh*_syn_ mutant lacking 3 conserved amino acids in the (p)ppGpp synthetase domain of the RSH (Rel) enzyme (25) was decreased and increased by guanosine/xanthosine and adenosine, respectively, similar to the wild-type (Table 1), further suggesting that these phenotypes are (p)ppGpp-independent.

**FIG 6:**
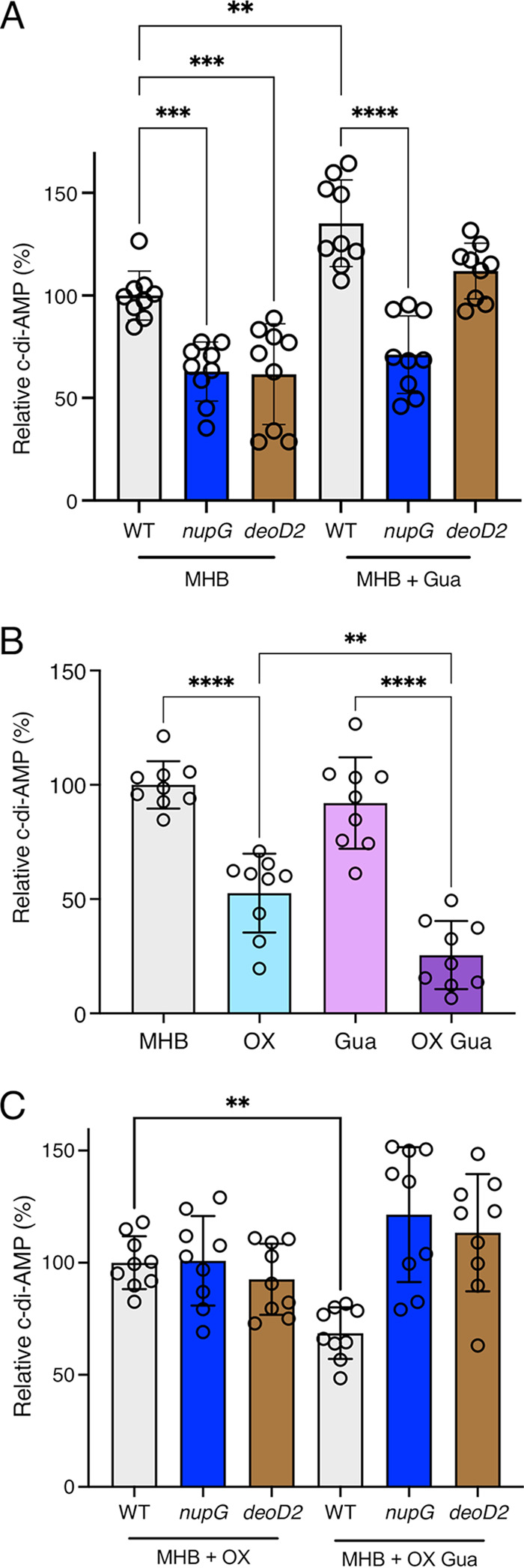
Exogenous guanosine controls c-di-AMP in a *nupG* and *deoD2*-dependent manner. (A) Relative c-di-AMP levels in wild-type, *nupG* and *deoD2* grown for 18 h in 5 mL cultures with and without 0.2 g/l guanosine (Gua). (B) Relative c-di-AMP levels in wild-type grown for 18 h in 12 mL cultures with and without 0.2 μg/mL oxacillin (OX) and/or 0.2 g/l Gua. (C) Relative c-di-AMP levels in wild-type, *nupG* and *deoD2* grown for 18 h in 12 mL cultures with and without 0.2 μg/mL Ox and/or 0.2 g/l Gua. Relative c-di-AMP levels are the averages of 9 biological replicates from a combination of 3 separate experiments with 3 bio-replicates each, normalized as % relative to wild type. Asterisks indicate statistically significant difference according to one-way ANOVA followed by Tukey’s multiple comparison *post hoc* test (*, *P* < 0.05; **, *P* < 0.01; ***, *P* < 0.001; ****, *P* < 0.0001). Error bars indicate standard deviation.

A competitive enzyme-linked immunosorbent assay (ELISA) revealed significantly reduced c-di-AMP levels in the *nupG* and *deoD2* mutants grown in MHB NaCl (Fig. 6A) without oxacillin supplementation, which may be keeping with the disruption to the purine salvage pathway caused by these mutations. Consistent with the putative role of NupG as a guanosine permease, exogenous guanosine (0.2 g/l) did not impact c-di-AMP production in the *nupG* mutant (Fig. 6A). Interestingly, exogenous guanosine did restore c-di-AMP to wild-type levels in the *deoD2* mutant (Fig. 6A), suggesting that even though this was not associated with any change in oxacillin susceptibility (Table 1), the combination of NaCl and imported guanosine leads to increased c-di-AMP production. This experiment was repeated using MHB media without 2% NaCl (Fig. S7), which also showed that c-di-AMP was reduced in the *nupG* and *deoD2* mutants, and that exogenous guanosine restored c-di-AMP to wild-type levels in both mutants. Collectively, these data reveal that *nupG* and *deoD2* mutations have robust impact on c-di-AMP in MHB and in MHB NaCl. However, the data suggest that in the absence of NaCl, and oxacillin, alternative permeases and nucleoside phosphorylases may substitute for the absence of NupG and DeoD2, respectively, and restore c-di-AMP regulation and homeostasis. Furthermore, NupG is a predicted sodium-nucleoside symporter, suggesting that, in the absence of sodium, it will not import its substrate nucleoside.

10.1128/mbio.02478-22.9 FIG S7 Relative c-di-AMP levels in wild-type, *nupG* and *deoD2* grown for 18 h in 5 mL MHB cultures without 2% NaCl, with and without 0.2 g/l guanosine (Gua). c-di-AMP levels are the averages of nine biological replicates from a combination of three separate experiments with 3 bio-replicates each, normalized as % relative to wild-type. Asterisks indicate statistically significant difference according to one-way ANOVA followed by Tukey’s multiple comparison post-hoc test (*, *P* < 0.05; **, *P* < 0.01; ***, *P* < 0.001; ****, *P* < 0.0001). Error bars indicate standard deviation. Download FIG S7, TIF file, 0.4 MB. Copyright © 2022 Nolan et al. 2022 Nolan et al. https://creativecommons.org/licenses/by/4.0/ This content is distributed under the terms of the Creative Commons Attribution 4.0 International license.

Next, the levels of c-di-AMP were measured in the wild-type, *nupG* and *deoD2* strains grown in MHB NaCl with OX, which are the same culture conditions used to measure OX susceptibility. Given the negative effect of OX/Gua combinations on growth, the culture volumes were scaled up for these experiments from 5 mL in 20 mL tubes (Fig. 6A) to 12 mL in 125 mL flasks (Fig. 6B). In contrast to growth in tubes (Fig. 6A), growth in larger cultures volumes and flasks did not reveal any increase in wild-type c-di-AMP levels in MHB NaCl supplemented with guanosine without OX (Fig. 6B), further showing that changes in growth conditions can lead to differentially regulated c-di-AMP levels in the cells. Importantly, the guanosine-induced OX susceptibility phenotype of the wild-type correlated with the lowest levels of c-di-AMP compared to growth in guanosine or OX alone (Fig. 6B). This significant effect was evident even when very low, sub-MIC (0.2 μg/mL) of OX used to minimize the effect on growth. In contrast, growth of the *nupG* and *deoD2* mutants in MHB supplemented with guanosine and OX increased c-di-AMP levels, albeit not reaching statistical significance (Fig. 6C). The reduced levels of c-di-AMP in MRSA cells exposed to the OX/Gua combination observed in this experiment is consistent with the significant increase in cell size observed under the same growth conditions (Fig. 5).

Exposure of JE2 cultures to adenosine alone reduced c-di-AMP levels, which were slightly reduced further by an OX/Ade combination compared to OX alone (Fig. S8A). Comparison of the impact of OX on relative c-di-AMP levels in the presence and absence of guanosine or adenosine further revealed that adenosine had no effect on c-di-AMP levels in the presence of OX (Fig. S8B), compared to the significant reduction in the presence of guanosine (Fig. S8C).

10.1128/mbio.02478-22.10 FIG S8 Relative c-di-AMP levels in MRSA cells exposed to adenosine. (A) Relative c-di-AMP levels in wild-type JE2 grown for 18 h in 5 mL MHB NaCl supplemented with OX 0.2 μg/mL or 0.2 g/l adenosine (Ade) or a combination of OX Ade. c-di-AMP levels are the averages of 9 biological replicates from a combination of 3 separate experiments with 3 bio-replicates each, normalized as % relative to wild-type. Asterisks indicate statistically significant difference according to one-way ANOVA followed by Tukey’s multiple comparison post-hoc test (*, *P* < 0.05; ***, *P* < 0.001; ****, *P* < 0.0001). Error bars indicate standard deviation. (B) Comparison of the impact of oxacillin on relative c-di-AMP levels in the presence and absence of guanosine. Relative c-di-AMP levels in MHB OX and MHB Gua/OX, compared to MHB and MHB Gua, respectively. (C) Comparison of the impact of oxacillin on relative c-di-AMP levels in the presence and absence of adenosine. Relative c-di-AMP levels in MHB OX and MHB Ade/OX were compared to MHB and MHB Ade, respectively. Download FIG S8, TIF file, 0.4 MB. Copyright © 2022 Nolan et al. 2022 Nolan et al. https://creativecommons.org/licenses/by/4.0/ This content is distributed under the terms of the Creative Commons Attribution 4.0 International license.

Consistent with an important role for c-di-AMP in these phenotypes, the increased OX resistance of a Δ*gdpP*::Km mutant (ANG 1961) in which c-di-AMP levels are increased (14) was unaffected by guanosine, xanthosine, or adenosine even at 1 g/l (Table 1). Similarly, the low OX MIC of a *dacA* G_206_S mutant in which c-di-AMP synthesis is impaired but not abolished (55) was reduced further by exogenous guanosine or xanthosine, and increased significantly by adenosine (Table 1). In summary, these data indicate that exogenous guanosine interferes with the normal activation of c-di-AMP production required for full expression of β-lactam resistance in MRSA.

## DISCUSSION

Identification and characterization of antibiotic adjuvants to maintain the therapeutic effectiveness of currently licensed antibiotics or facilitate the reintroduction of drugs for which resistance is widespread, is a promising part of efforts to address the AMR crisis. In keeping with previous studies implicating purine metabolism in the regulation of MRSA β-lactam resistance (36, 56), we report here that mutations interfering with the purine salvage and biosynthetic pathways increased oxacillin resistance. These data prompted us to evaluate if the opposite was true and if susceptibility to β-lactams could be increased by exposing MRSA to exogenous purine nucleotides and nucleosides. Guanosine and xanthosine, which are fluxed through the GTP branch of purine biosynthesis reduced the oxacillin MIC of JE2 into the susceptibility range, in a dose dependent manner, whereas adenosine, which is fluxed to ATP, significantly increased oxacillin resistance. Furthermore, inosine, which can be fluxed to ATP or GTP, had almost no effect. These data reveal an important role for purine homeostasis in controlling the susceptibility of MRSA to β-lactam antibiotics and add to several recent studies implicating the regulation of purine biosynthesis in the control of virulence (27, 57, 58). Interestingly, as part of a 2017 study from our laboratory showing that β-lactams can attenuate MRSA virulence, transcriptomic analysis revealed that, after the *agr* locus, the *pur* operon was the second most downregulated gene cluster during growth in oxacillin (42). Furthermore, oxacillin-induced repression of the *pur* operon was accompanied by significantly increased expression of *purR* (42), a finding in keeping with the subsequent reports that PurR also acts as a repressor of virulence (57, 58). Taken together, it appears that under β-lactam stress, MRSA may attempt to limit flux through purine biosynthetic pathways as a means of increasing resistance and that, conversely, increased guanosine or xanthosine flux into the GTP branch of purine metabolism has the reverse effect and reduces β-lactam resistance.

A recent paper describing the interplay between *de novo* purine biosynthesis and c-di-AMP signaling demonstrated that a *purF* mutation was associated with reduced levels of c-di-AMP (27). Here, we report that, with the exception of *purH*, mutations in all other *pur* operon genes including *purF* were accompanied by increased β-lactam resistance. The reduction in c-di-AMP levels in a *purF* mutant reported by Li et al. (27) and the increase in oxacillin resistance described in this study may at first appear to be contradictory. However, it is important to note that the contribution of purine homeostasis to β-lactam resistance can only be accurately characterized when MRSA is grown in the presence of these antibiotics. Thus, under standard growth conditions (MHB NaCl media), our data showed that the purine salvage pathway *nupG* and *deoD2* mutations were associated with reduced levels of c-di-AMP, whereas under very mild oxacillin stress, c-di-AMP was restored to wild-type levels in these mutants. Furthermore, the reduced levels of c-di-AMP in wild-type exposed to the guanosine/oxacillin combination was reversed in the *nupG* and *deoD2* mutants.

Among the purine metabolism mutations examined, *purH* and *deoD1* were not found to have any impact on β-lactam resistance, although both mutants remained susceptible to OX/Gua treatment. Purine nucleoside phosphorylases typically form oligomers raising the possibility that DeoD1 and DeoD2 can heterodimerise, which may influence their contribution to purine salvage and β-lactam resistance, particularly if they are differentially regulated under specific growth conditions. The *deoD1* gene is co-located on the chromosome with a major facilitator superfamily (Mfs) gene, *deoC1* (deoxyribose-phosphate aldolase), and *deoB* (phosphopentomutase), which may be expressed as an operon. Mutations in the Mfs gene, *deoC1* and *deoB* genes alone were not associated with altered OX susceptibility (59). Nevertheless, DeoD1 activity may be dependent on the Mfs protein, or a solute/ion transported by this membrane protein. In contrast, the *deoD2* gene is monocistronic and a second deoxyribose-phosphate aldolase, *deoC2*, sharing 98% identity with *deoC1*, is transcribed divergently from *deoD2*. The *purH* gene, which encodes the bifunctional phosphoribosylaminoimidazolecarboxamide formyltransferase/IMP cyclohydrolase is the penultimate gene in the *purEKCSQLFMNHD* operon followed only by *purD*. A *purD* mutation, like all other *pur* operon mutations is accompanied by increased OX resistance. PurH is responsible for the final step in the purine biosynthetic pathway, converting 5-aminoimidazole-4-carboxamide ribonucleotide (AICAR) into IMP. AICAR is produced by PurB, which is also part of the purine salvage pathway and *purB* can be transcriptionally regulated independent of the *pur* operon (60). Unlike other Pur enzymes, the bifunctional activity of PurH (synthesis and break-down of IMP), combined with its requirement for AICAR from the *de novo* or purine salvage pathways points to a complex role of this enzyme in purine metabolism and may suggest that its impact on OX resistance is more dependent on growth conditions than other *pur* operon enzymes.

Arising from studies to uncover the therapeutic potential of bactericidal and bacteriostatic compounds produced by coagulase-negative staphylococci (CoNS) to compete with S. aureus in shared ecological niche, the Heinrich’s group identified the CoNS-produced purine analogue 6-thioguanine (6-TG) and revealed its potential to both inhibit growth of S. aureus by interfering with ribosome biogenesis and downregulate *agr*-controlled virulence under purine limited growth conditions (61). Resistance to 6-TG can arise through mutations in PbuG (also named StgP, 6-thioguanine permease) and the Hpt hypoxanthine phosphoribosyltransferase (45), demonstrating that guanine/6-TG are transported and metabolized by PbuG/StgP and Hpt, respectively. We showed here that guanosine was unable to resensitize the 6-TG resistant *hpt* mutants R10 and R11 (45) to OX, further supporting the role for Hpt in guanine/6-TG metabolism. Our screen of suppressor mutants resistant to OX/Gua combinations also identified stop codon mutations in *hpt* as well as *nupG*, *prs*, *rpoB*, several tRNA genes, and *clpX*. Interestingly, while the R10 and R11 *hpt* mutants were resistant to guanosine/OX combinations, they were susceptible to OX/Xan, presumably because xanthosine is processed by the Xpt phosphoribosyltransferase thus bypassing Hpt. On the other hand, the GS8 and GS25 OX/Gua suppressor mutants carrying a Hpt L_113_Stop mutation unexpectedly remained resistant to OX/Xan. Although the reason for this difference is unclear, one possibility is that Hpt also plays a role in processing xanthosine. If this is true, the Hpt amino acid substitutions in R10 and R11 must interfere with guanosine processing, but may not affect processing of xanthosine whereas the L_113_Stop mutation presumably blocks processing of both nucleosides. Interestingly, a M_1_I mutation in *hpt*, likely to negatively impact translation initiation, was previously implicated by the HoR phenotype (36). Similarly, a *guaA* deletion mutation resulted in HoR resistance. There are no transposon insertions in *hpt* or *guaA* in the NTML, indicating that these genes are important for growth under standard laboratory conditions. Collectively, our data are consistent with previous studies, indicating that mutations negatively impacting GTP biosynthetic pathway enzyme activity are accompanied by increased resistance to β-lactams and β-lactam/nucleoside combinations.

Our data showed that *nupG* and *deoD2* mutants significantly increased β-lactam resistance independent of changes in PBP2a levels. Several lines of evidence support the conclusion that purine homeostasis regulated β-lactam resistance is mechanistically separate from PBP2a expression. For instance, our data showed that guanosine also significantly reduces the OX MICs of a laboratory MSSA strain and the NTML *mecA* transposon mutant. Two *clpX* stop codon mutations (G_98_* and G_278_*) were also identified giving rise to resistance to OX/Gua and OX/Xan combinations, and very high OX MICs. The ClpXP chaperone-protease complex is known to control β-lactam resistance in MRSA (17, 18, 62), independent of changes in *mecA* transcription or PBP2a levels (17). Here, we also identified RpoB T_480_K and T_622_I substitutions implicated in resistance to nucleoside/OX combinations. Two independent studies of *rpoB* and *rpoC* mutations associated with high-level β-lactam resistance ruled out changes in PBP2a expression (16, 56). In a recent analysis, Giulieri et al. (63) implicated both *gdpP*/*dacA*-regulated c-di-AMP signal transduction, as well as the ClpXP chaperone-protease complex in *mecA*-independent, low level OX resistance. Collectively, these findings support the conclusion that altered regulation of PBP2a expression does not play an important role in the regulation of β-lactam susceptibility by nucleoside/OX combinations. Instead, our data showing that guanosine alone, and in particular in combination with OX, significantly reduces c-di-AMP levels in a NupG/DeoD2-dependent manner and dramatically increases cell size. Osmoregulation and cell size are among the important phenotypes controlled by c-di-AMP (14, 25) suggesting that deregulation of cellular osmotic control by exogenous purine nucleosides is the most likely mechanism for increased β-lactam susceptibility.

From a therapeutic perspective, our *in vitro* kill curves revealed significant bactericidal activity using combinations of guanosine and OX at concentrations up to 128 μg/mL, which are within the range of flucloxacillin intravenously administered to human patients (100-200 mg/kg/day). While the emergence of OX/Gua suppressor mutants appeared to be significantly reduced at higher oxacillin concentrations (as evidenced by the absence of re-growth in kill curve cultures), this remains a potential barrier to the use of nucleosides as β-lactam adjuvants. Future studies to measure the anti-MRSA activity of different purine nucleosides or purine analogues in combination with different β-lactams may identify more effective therapeutic regimes. Nevertheless, drugs derived from purine and pyrimidine nucleotides are already used for the treatment of cancer and viral infections highlighting the potential clinical applications of these findings, not only for MRSA but also other pathogens on the World Health Organization's list of priority antibiotic-resistant pathogens. Using purine nucleosides as adjuvants to increase the susceptibility of clinically important pathogens to β-lactams has the potential to facilitate new treatments with lower antibiotic doses and with drug combinations that are toxic at higher concentrations.

## MATERIALS AND METHODS

### Bacterial strains and growth conditions.

Bacterial strains and plasmids used in this study are listed in Table S1. Escherichia coli strains were grown in Luria Bertani (LB) broth or agar (LBA) and S. aureus strains were grown in MHB, Mueller-Hinton Agar (MHA), Tryptic Soy Broth (TSB), Tryptic Soy Agar (TSA), chemically defined medium (CDM), supplemented with erythromycin (Erm) 10 μg/mL, chloramphenicol (Cm) 10 μg/mL, ampicillin (Amp) 100 μg/mL, kanamycin (Km) 90 μg/mL, guanosine, xanthosine, or adenosine (0.2 to 1 g/l) where indicated.

Data from growth experiments in a Tecan Sunrise microplate instrument were recorded using Magellan software. Overnight cultures were grown in 5 mL TSB using a single colony, and were washed once in PBS before being used to inoculate CDM cultures. PBS washed overnight cultures were adjusted to an OD_600_ of 0.1 in PBS and 10 μL inoculated into 200 μL media per well before being incubated at 35°C for 24 h with shaking and *A*_600_ recorded every 15 min intervals.

For flask growth curves, 250 mL flasks were filled with 25 mL growth media, and cultures grown overnight in TSB were used to inoculate the media at a starting *A*_600_ of 0.01. Flasks were incubated at 37°C shaking at 200 rpm. *A*_600_ readings were measured at 2 h intervals for 12 h, and an extra reading was taken at 24 h.

CFU were enumerated by serially diluting 100 μL aliquots that were removed from flask cultures. Plates were incubated for 20 h at 37°C and colonies enumerated, and CFU per mL suspension per OD_600_ were calculated. Three independent biological replicates were performed for each strain, and the resulting data plotted using GraphPad Prism software.

### Genetic manipulation of S. aureus.

Phage 80α was used as described previously (64) to transduce the transposon insertions from NE650, NE1419 into the parent JE2 strain to rule out the possibility that uncharacterized mutations in the NTML mutants may contribute to the increased antibiotic resistance phenotypes. Transposon insertions were verified by PCR amplification of the target loci using primers listed in Table S2.

10.1128/mbio.02478-22.2 TABLE S2 Oligonucleotide primers used in this study. Download Table S2, DOCX file, 0.02 MB. Copyright © 2022 Nolan et al. 2022 Nolan et al. https://creativecommons.org/licenses/by/4.0/ This content is distributed under the terms of the Creative Commons Attribution 4.0 International license.

To complement NE650 and NE1419, 1,387bp and 1,740bp PCR fragments were amplified using JE2 gDNA as template and the primers INF_deoD_F/INF_deoD_R and INF_nupG_F/INF_nupG_R, respectively (Table S2 and Fig. S4). The Clontech Infusion Cloning kit was used to insert the fragments into the *Eco*RI restriction site of pLI50 to generate pLI50_*nupG*, and pLI50_*deoD2*. The recombinant plasmids were first transformed into cold-competent E. coli HST08 before being transformed by electroporation into the restriction-deficient S. aureus strain RN4220, and finally into NE650 and NE1419.

### PBP2a Western blot analysis.

Single colonies from wild-type JE2, NE1419 (*nupG*), NE650 (*deoD2*), NE1419 pLI50_*nupG*, NE650 pLI50_*deoD2*, MSSA strain 8325-4 (negative control), and HoR MRSA strain BH1CC (positive control) were inoculated in MHB 2% NaCl overnight and grown at 35°C with 200 rpm shaking. All cultures were supplemented with 5 μg/mL OX, with the exception of 8325-4 which was grown in MHB 2% NaCl without antibiotic and BHICC which was grown with 32 μg/mL OX. The next day, samples were pelleted and resuspended in PBS to an *A*_600_ = 10. Six microliters of lysostaphin (10 μg/mL) and 1 μL of DNase (10 μg/mL) was added to 500 μL of this concentrated cell suspension before being incubated at 37°C for 40 min. Next, 50 μL of 10% SDS was added, and the incubation continued for a further 20 min. The lysed cells were then pelleted in a microcentrifuge for 15 min, following which the protein-containing supernatant was collected and total protein concentration determined using the Pierce BCA Protein assay kit. For each sample, 6 μg total protein was run on a 7.5% Tris-Glycine gel, transferred to a polyvinylidene difluoride (PVDF) membrane, and probed with anti-PBP2a (1:1000), followed by HRP-conjugated protein G (1:2000) and colorimetric detection with Opti-4CN Substrate kit. Three independent experiments were performed.

### Antibiotic disk diffusion susceptibility assays.

Disk diffusion susceptibility testing was performed in accordance with Clinical and Laboratory Standards Institute (CLSI) guidelines (65) with the following modifications: Overnight cultures were diluted into fresh TSB and grown for 3 h at 37°C. Day cultures were then adjusted to *A*_600_ = 0.5 and 150 μL of this suspension was swabbed evenly 3 times across the surface of an MHA plate (4 mm agar depth). Then, 6 mm antibiotic disks were applied, before incubation for times specified by CLSI guidelines for stated antibiotics at 35 to 37°C. Twenty microliters of guanosine at varying concentrations (50 to 750 μg/mL) was spotted onto disks to demonstrate synergistic activity with β-lactam antibiotics where indicated. Three independent measurements were performed for each strain.

### Antibiotic MIC and synergy assays.

MIC measurements by broth microdilutions were performed in accordance with CLSI methods for dilution susceptibility testing of staphylococci (66) with modifications as follows: guanosine, xanthosine, or adenosine were supplemented into culture media at concentrations ranging from 200 to 1000 μg/mL. Strains were first grown at 37°C on MHA (or MHB NaCl for OX MIC assays) for 24 h and 5 to 6 colonies were resuspended in 0.85% saline before being adjusted to 0.5 McFarland standard (*A*_600_ = 0.1). The cell suspension was then diluted 1:20 in 0.85% saline and 10 μL used to inoculate 100 μL MHB/MHB NaCl supplemented with purines as indicated in the individual wells of 96-well plates. The plates were incubated at 37°C for 24 h and MIC values were recorded as the lowest antibiotic concentration where no growth was observed.

### Isolation of suppressor mutants resistant to guanosine/oxacillin combination.

Suppressor mutants were isolated from JE2 cultures serially passaged in MHB 2% NaCl supplemented with 200 μg/mL guanosine and 32 μg/mL OX at 35°C. Using 96-well plates, 100 μL of culture medium was inoculated with 2 to 8 × 10^5^ CFU/mL from a JE2 overnight culture and growth was monitored for 24 h in a Tecan Sunrise microplate instrument using Magellan software, after which a 1:20 dilution was used to inoculate 100 μL of the same medium in the corresponding well of a new plate, which was incubated for a further 24 h. In wells showing visible growth, 5 μL of culture was inoculated onto TSA plates to purify single colonies from the suppressor strains. The susceptibility (MICs) of each of the isolated suppressor strains to OX and OX/GUA combinations was confirmed as described above.

### Genomic DNA extraction and whole-genome sequencing.

Genomic DNA (gDNA) extractions were performed using the Wizard Genomic DNA purification kit (Promega) following pretreatment of S. aureus cells with 10 μg/mL lysostaphin (Ambi Products LLC) at 37°C for 30 min. DNA libraries were prepared using an Illumina Nextera DNA kit. An Illumina MiSeq v2 300 cycle kit was used for the genome sequencing and the 150 bp paired end sequencing run was performed at the MRC London Institute of Medical Sciences Genomics Facility. The CLC Genomics Workbench software (Qiagen Version 20) was used for genome sequencing analysis of the different strains, as described previously (31). As a reference genome, a contig was produced for wild-type JE2 by mapping Illumina reads onto the closely related USA300 FPR3757 genome sequence (RefSeq accession number NC_07793.1). The Illumina short read sequences from NTML mutants of interest were then mapped onto the assembled JE2 sequence, and the presence of the transposon insertion confirmed. Single Nucleotide Polymorphisms (SNPs), deletions or insertions were mapped to the genomes of the NTML mutants and OX/Gua resistant suppressor strains and the presence of large deletions ruled out by manually searching for zero coverage regions. Whole-genome sequence data is available from the European Nucleotide Archive (Study Accession number PRJEB55671), accession numbers (ERS13358424 - ERS13358446).

### Antibiotic kill curves.

Kill Curves were performed according to CLSI guidelines to determine the extent of synergy between purines and oxacillin. Overnight cultures grown in MHB NaCl were diluted 1:100 and allowed to grow for 3 h (exponential growth phase) before being inoculated into 25 mL of MHB NaCl in 250 mL flasks at a starting cell density of approximately 1 × 10^6^ CFU/mL. The flasks were incubated at 35°C with shaking at 200 rpm and CFU were enumerated after 0, 2, 4, 6, 8, 12, and 24 h by serially diluting and plating 100 μL aliquots removed from flask cultures on tryptone soya agar plates.

### Microscopy and cell size determination.

For microscopy experiments, MRSA strain JE2 was grown overnight at 37°C in MHB. The next day, it was back-diluted into MHB, MHB Gua 0.2 g/l, MHB OX 1 μg/mL, MHB Gua 0.2 g/l OX 1 μg/mL, MHB Ade 0.2 g/l, MHB Ade 0.2 g/l OX 1 μg/mL and day cultures were grown for 5 h at 35°C. After the 5 h growth, the day cultures were washed and normalized to an *A*_600_ of 1 in PBS and 75 μL of these cultures were double stained for 30 min at 37°C with vancomycin-BODIPY FL at a final concentration of 2 μg/mL and WGA Alexa Fluor 594 at a final concentration of 25 μg/mL. Bacteria were then collected by centrifugation for 2 min at 14,000 × *g*. The cells were resuspended with 100 μL of PBS, pH 7.4, and 5 μL of this sample was spotted onto a thin 1% agarose gel patch prepared in PBS. Stained bacteria were then imaged at ×1000 magnification using an Olympus LS FLUOVIEW Fv3000 Confocal Laser Scanning Microscope. Cell size was measured as previously described (31) using ImageJ software. Images of cells from 3 biological replicates were acquired, 40 cells measured per biological replicate (120 cells in total per condition) for Fig. 5B, and 50 cells measured per biological replicate (150 cells in total per condition) for Fig. S5B, and the average and standard deviations for the 3 biological replicates were plotted using GraphPad Prism version 9.2 and significant differences were determined using one-way ANOVA followed by Tukey’s *post hoc*.

10.1128/mbio.02478-22.7 FIG S5 Exposure to adenosine does not increase MRSA cell size. (A) Representative microscopic images of JE2 cells grown in MHB NaCl or MHB NaCl supplemented with adenosine (Ade, 0.2 g/l), oxacillin (OX,1 μg/mL) or a combination of Ade and OX labeled with vancomycin-BODIPY FL (green, top panel) or WGA Alexa Fluor 594 (red, bottom panel). (B) Average diameter of JE2 cells grown in MHB NaCl or MHB NaCl supplemented with adenosine (Ade) (0.2 g/l), oxacillin (0.2 μg/mL) or a combination of OX Ade. Images of cells from 3 biological replicates were acquired using Fv3000 confocal microscope and software, 50 cells measured per biological replicate (150 cells in total per growth condition), and the average for the 3 biological replicates were plotted using GraphPad Prism V9. Asterisks indicate statistically significant difference according to one-way ANOVA followed by Tukey’s multiple comparison post-hoc test (****, *P* < 0.0001). Error bars indicate standard deviation. Download FIG S5, TIF file, 1.1 MB. Copyright © 2022 Nolan et al. 2022 Nolan et al. https://creativecommons.org/licenses/by/4.0/ This content is distributed under the terms of the Creative Commons Attribution 4.0 International license.

### GTP assays.

A GTPase-Glo bioluminescence assay kit (Promega) was used, and the manufacturer’s guidelines were adjusted to measure relative intracellular GTP levels by luminescence. Briefly, overnight cultures of JE2, *nupG*, and *deoD2* grown at 37°C in MHB NaCl were used to inoculate duplicate MHB NaCl cultures supplemented with guanosine (Gua, 0.2 g/l) and/or OX (OX, 1 μg/mL) at a starting *A*_600_ = 0.05 before these were again incubated overnight at 37°C. Cell pellets were lysed, and total protein concentrations determined using a Bicinchoninic Acid (BCA) assay. The protein concentration in each lysate was adjusted to 50 μg/mL and 2.5 μL (125 ng) used in each GTPase-Glo assay.

### Measurement of (p)ppGpp.

Cultures of JE2, *nupG*, and *deoD2* were grown overnight at 37°C in MHB, followed by dilution in MHB NaCl with OX (1 μg/mL) and/or guanosine (0.2 g/l) to *A*_600_ = 0.05. Cells were grown to *A*_600_ = 0.5 and divided into 2 aliquots. To one, 3.7 MBq of (^32^P)H_3_PO_4_ was added, while the other lacked radiation to allow for normalization of cultures via protein concentrations by BCA assay. Both sets of culture were grown overnight, and the non-radioactive set used to determine protein concentration. Radioactive culture corresponding to 150 ng of protein was lysed by the addition of 2 M formic acid and subsequently subjected to 4 freeze/thaw cycles. Ten microliters of the supernatant fractions were subsequently spotted on PEI-cellulose F TLC plates (Merck Millipore), and nucleotides were separated using a 1.5 M KH_2_PO_4_, pH 3.6, buffer. The radioactive spots were visualized using an FLA 7000 Typhoon PhosphorImager, and data were quantified using ImageQuantTL software.

### Quantification of c-di-AMP levels using a competitive ELISA.

Relative intracellular levels of c-di-AMP were determined using a previously described competitive ELISA method (67) with some modifications. Briefly, single colonies of wild-type JE2, *nupG* (NE1419), and *deoD2* (NE650) were picked from TSA plates and used to inoculate 5 mL or 12 mL of MHB 2% NaCl with or without guanosine (0.2 g/l) or adenosine (0.2 g/l) and/or OX (0.2 μg/mL) and the cultures were incubated for 18 h at 37°C with shaking. The 5 mL cultures were grown in 20 mL tubes and 12 mL cultures were grown in 125 mL flasks. The next day, 1.5 mL (for all strains grown without OX), 3 mL (for *nupG* and *deoD2* grown in OX and the wild-type grown in OX alone), or 6 mL (for wild-type JE2 grown in both OX and guanosine) were collected by centrifugation, washed three times with PBS, resuspended in 0.75 mL of 50 mM Tris pH 8 buffer supplemented with 20 ng/mL lysostaphin and incubated for 1 h before completing cell lysis by bead beating. Following centrifugation for 5 min at 17,000 × *g*, the supernatant from the cell lysates were transferred to new tubes and protein concentration of the samples was determined using a Pierce BCA protein assay kit (Thermo Scientific). The samples were then heated to 95°C for 10 min, centrifuged for 5 min at 17,000 × *g*, and the supernatant containing c-di-AMP was transferred to a new tube. The samples were diluted to a protein concentration of 300 to 800 μg/mL, as appropriate. A total of 100 μL of coating buffer (50 mM Na_2_C0_3_, 50 mM NaHCO_3_, pH 9.6) containing 10 μg/mL of the c-di-AMP binding protein CpaA_SP_ was aliquoted into each well of a 96-well Nunc MaxiSorp plate (Thermo Scientific), which was then incubated for 18 h at 4°C. The plate was then washed three times with 200 μL PBST pH 7.4 (10 mM Na_2_HPO_4_, 1.8 mM KH_2_PO_4_ 137 mM NaCl, 2.7 mM KCl, 0.05% [vol/vol] Tween 20), blocked for 1 h at 18°C with 150 μL blocking solution (1% BSA in PBS pH 7.4), and, again, washed three times with 200 μL PBST. A total of 250 μL aliquots of the samples (with 3 technical replicates for each biological replicate) or standards (2 technical replicates) were then mixed with 250 μL of a 50 nM biotinylated c-di-AMP solution prepared in 50 mM Tris pH 8 buffer. For the standard curve, c-di-AMP standards were prepared in 50 mM Tris pH 8 buffer at concentrations of 0, 12.5, 25, 37.5, 50, 75, 100, and 200 nM. Following the addition of the samples and the standards, the 96-well plates were incubated for 2 h at 18°C, and then washed three times with PBST. Next, 100 μL of a high-sensitivity streptavidin-HRP solution (Thermo Scientific) diluted 1:500 in PBS was added to each well and the plate was incubated for 1 h at 18°C. The plate was washed three times with 200 μL PBST, and 100 μL of a developing solution (0.103 M NaHPO_4_, 0.0485 M citric acid, 500 mg/L o-phenylenediamine dihydrochloride, 0.03% H_2_O_2_) was added to each well and the plate incubated for 15 min at 18°C. The reaction was stopped by adding 100 μL of 2 M H_2_SO_4_ solution and the absorbance measured in a plate reader at 490 nm. c-di-AMP concentrations were calculated as ng c-di-AMP/mg protein. The data from 3 independent experiments was collated and presented by showing relative c-di-AMP levels in all samples compared to the wild-type grown in MHB NaCl, MHG NaCl Ox or MHB NaCl Ox/Gua as appropriate, which was assigned a value of 100%. Standard deviations are shown, and significant differences were determined using one-way ANOVA followed by Tukey’s *post hoc* tests.

### Data availability.

Whole-genome sequence data is available from the European Nucleotide Archive (Study Accession number PRJEB55671), accession numbers ERS13358424 - ERS13358446. The SAUSA300_FRP3757 (TaxID: 451515) reference genome sequence is available from NCBI.
