# Supplementary figures and images for: Purine Nucleosides Interfere with c-di-AMP Levels and Act as Adjuvants To Re-Sensitize MRSA To β-Lactam Antibiotics
Source: mBio. 2022 Dec 12;14(1):e02478-22. doi: 10.1128/mbio.02478-22 (PMC9973305; doi:10.1128/mbio.02478-22)

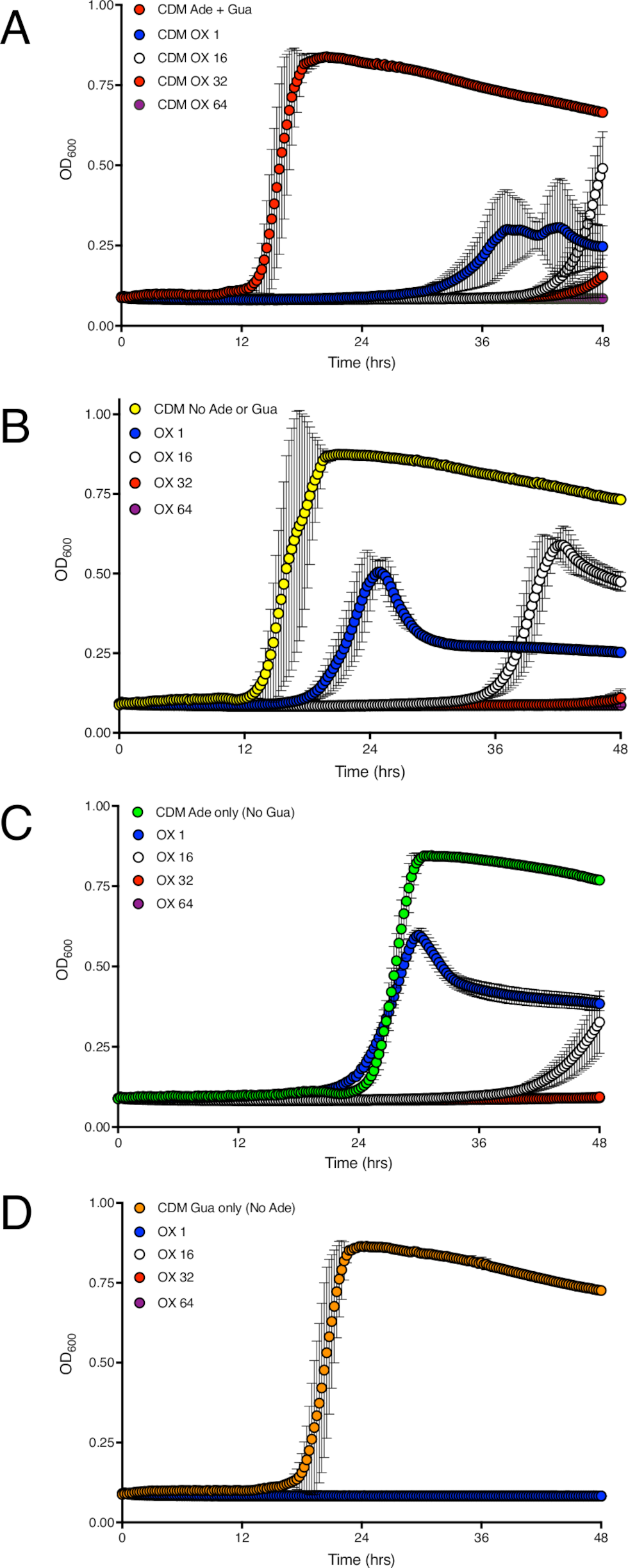

Supplement: FIG S1 [file mbio.02478-22-s0001.tif]

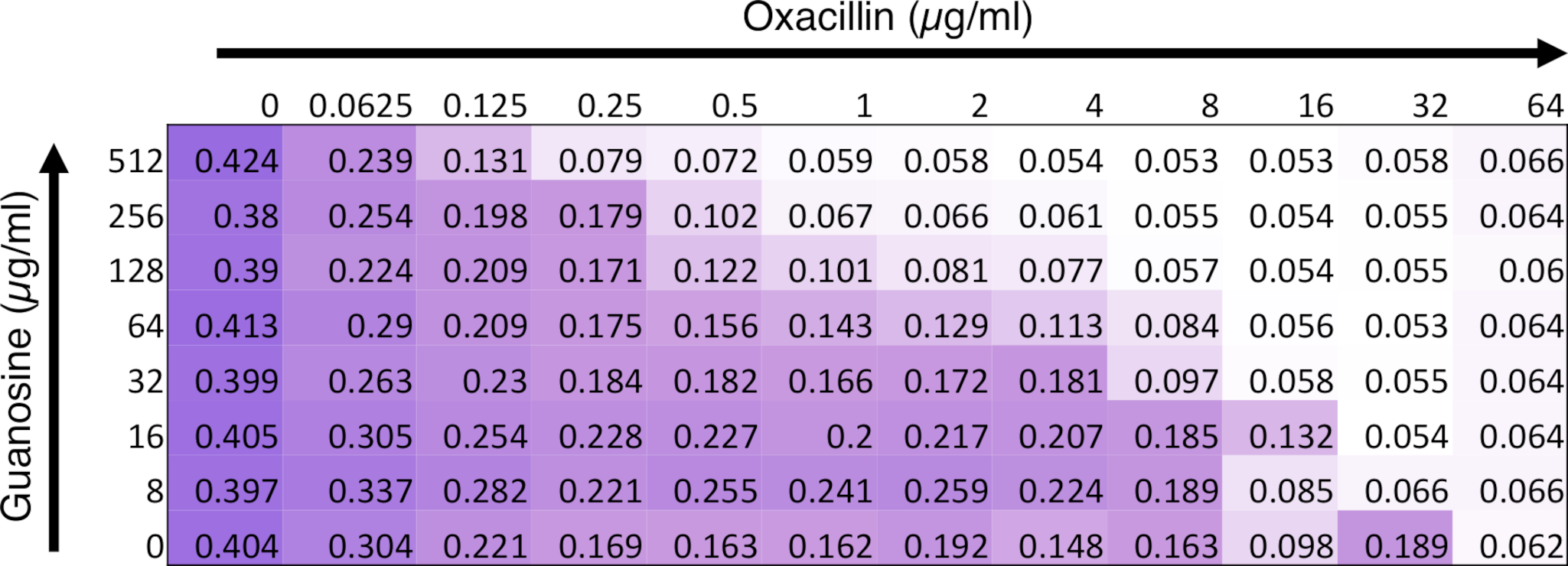

Supplement: FIG S2 [file mbio.02478-22-s0002.tif]

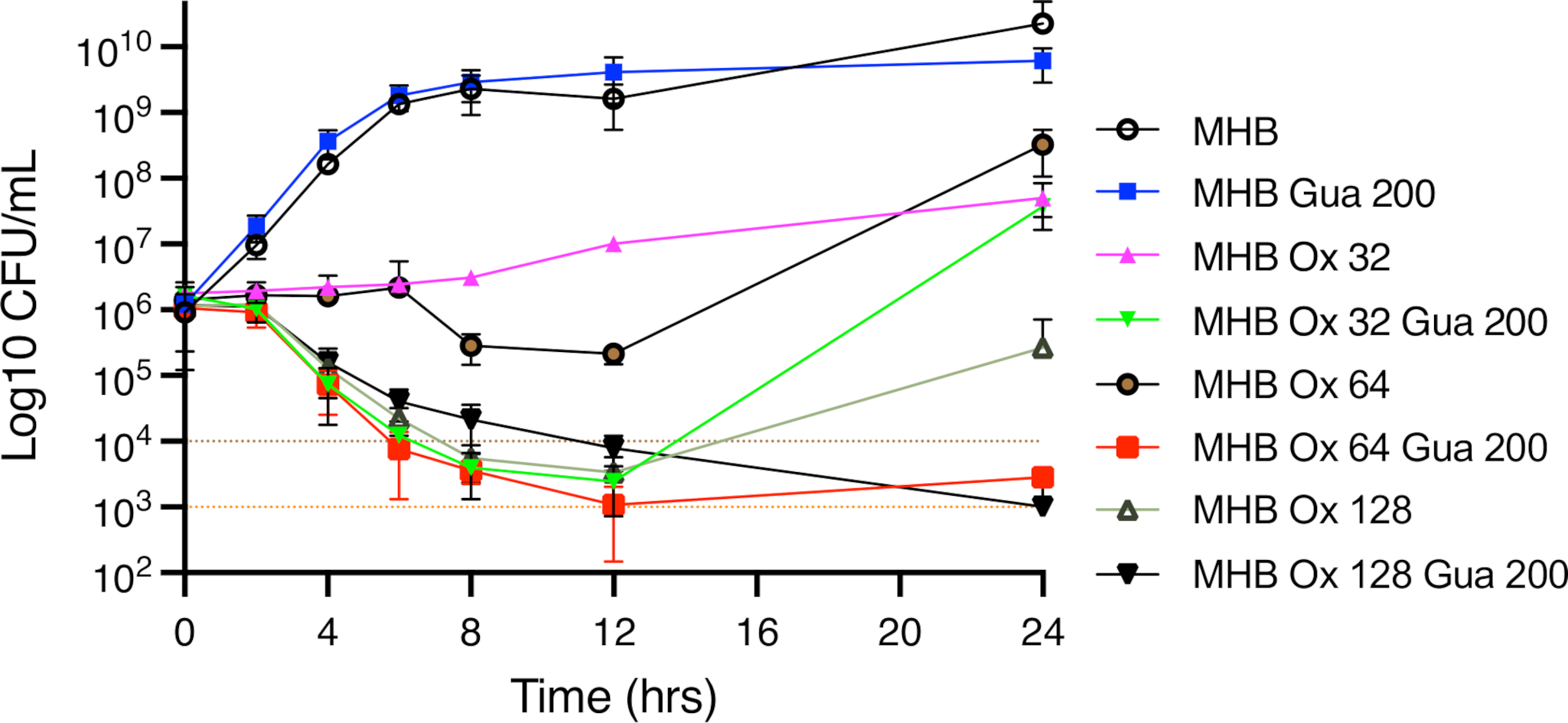

Supplement: FIG S3 [file mbio.02478-22-s0003.tif]

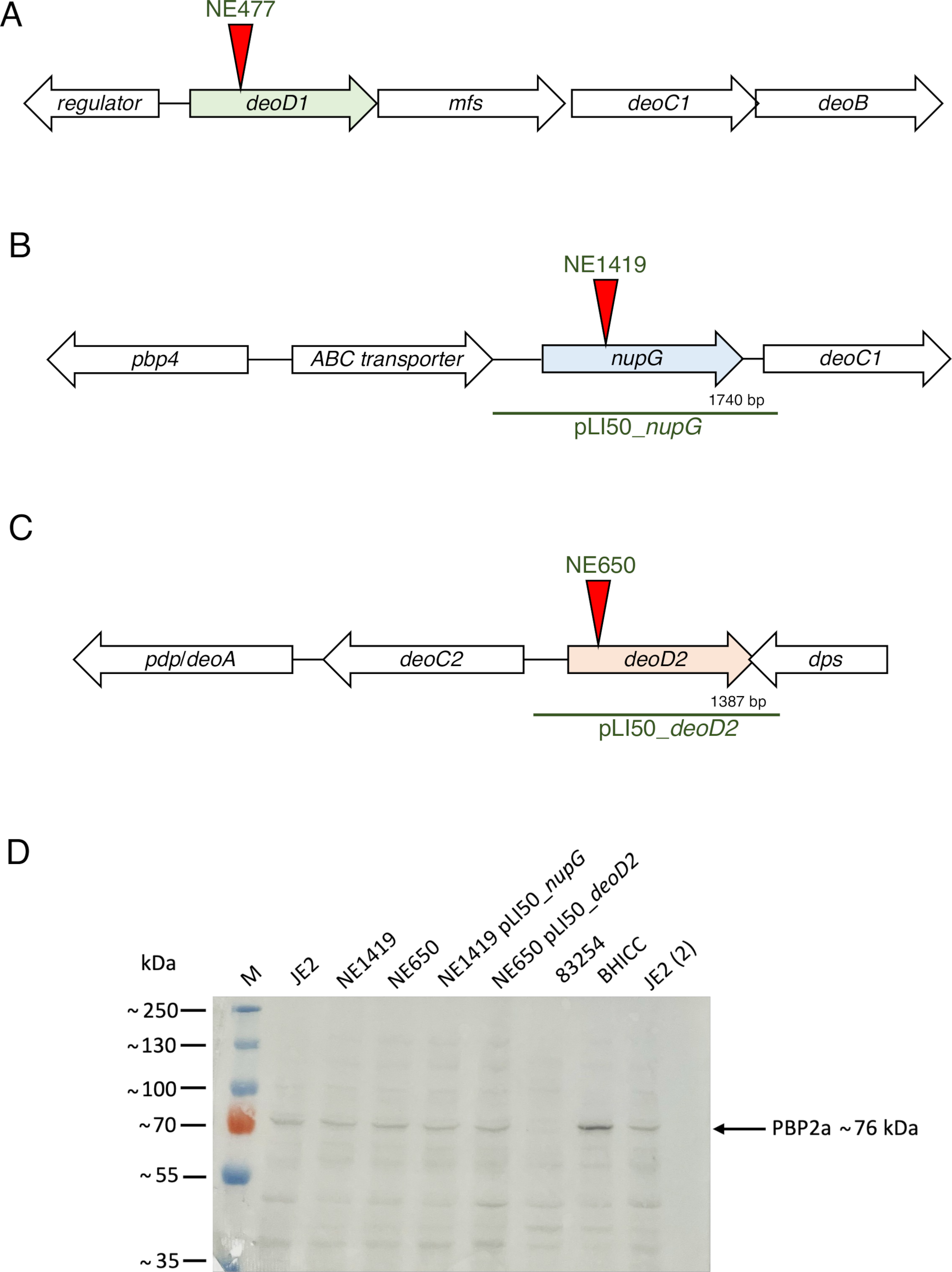

Supplement: FIG S4 [file mbio.02478-22-s0004.tif]

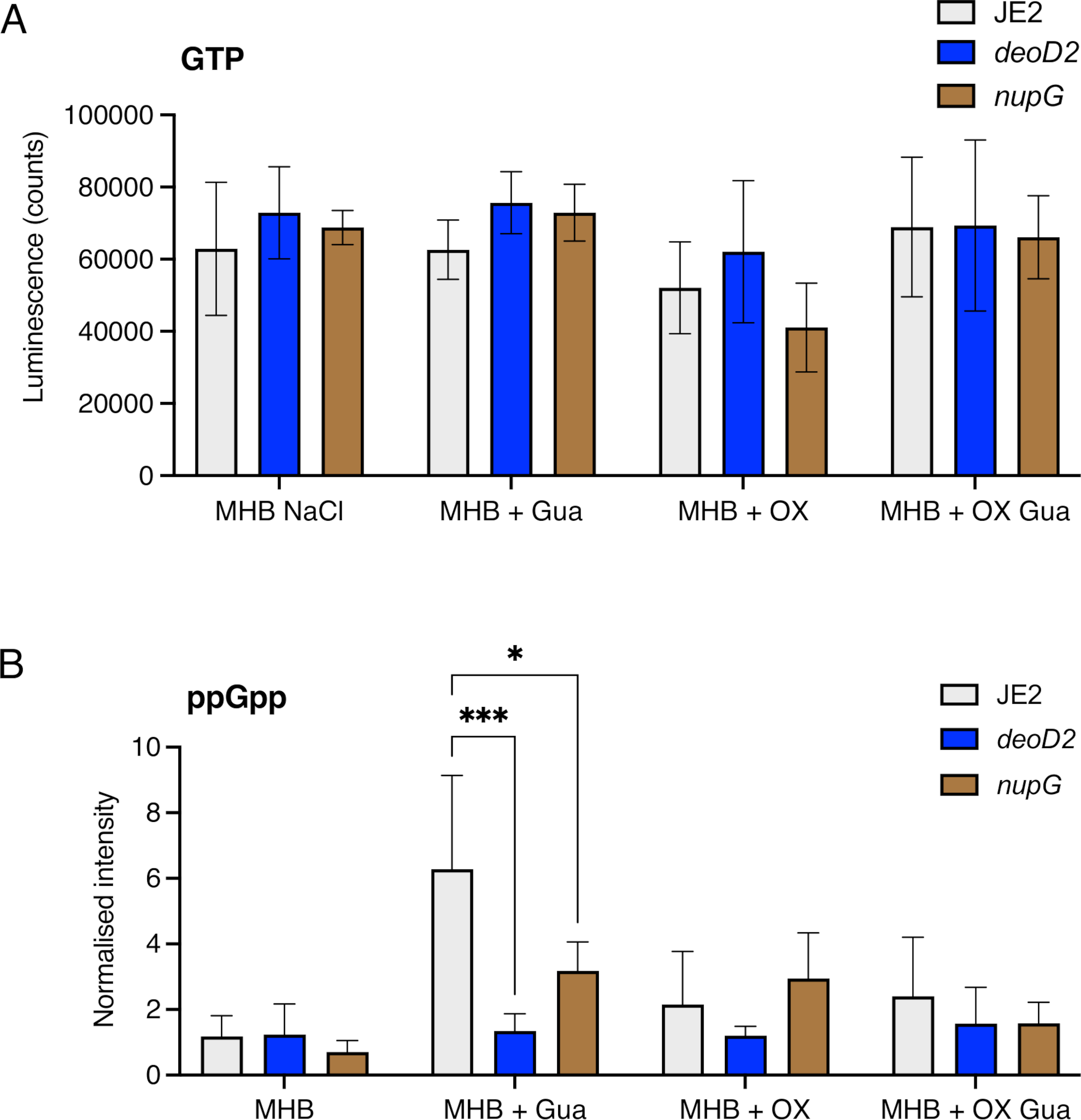

Supplement: FIG S6 [file mbio.02478-22-s0006.tif]

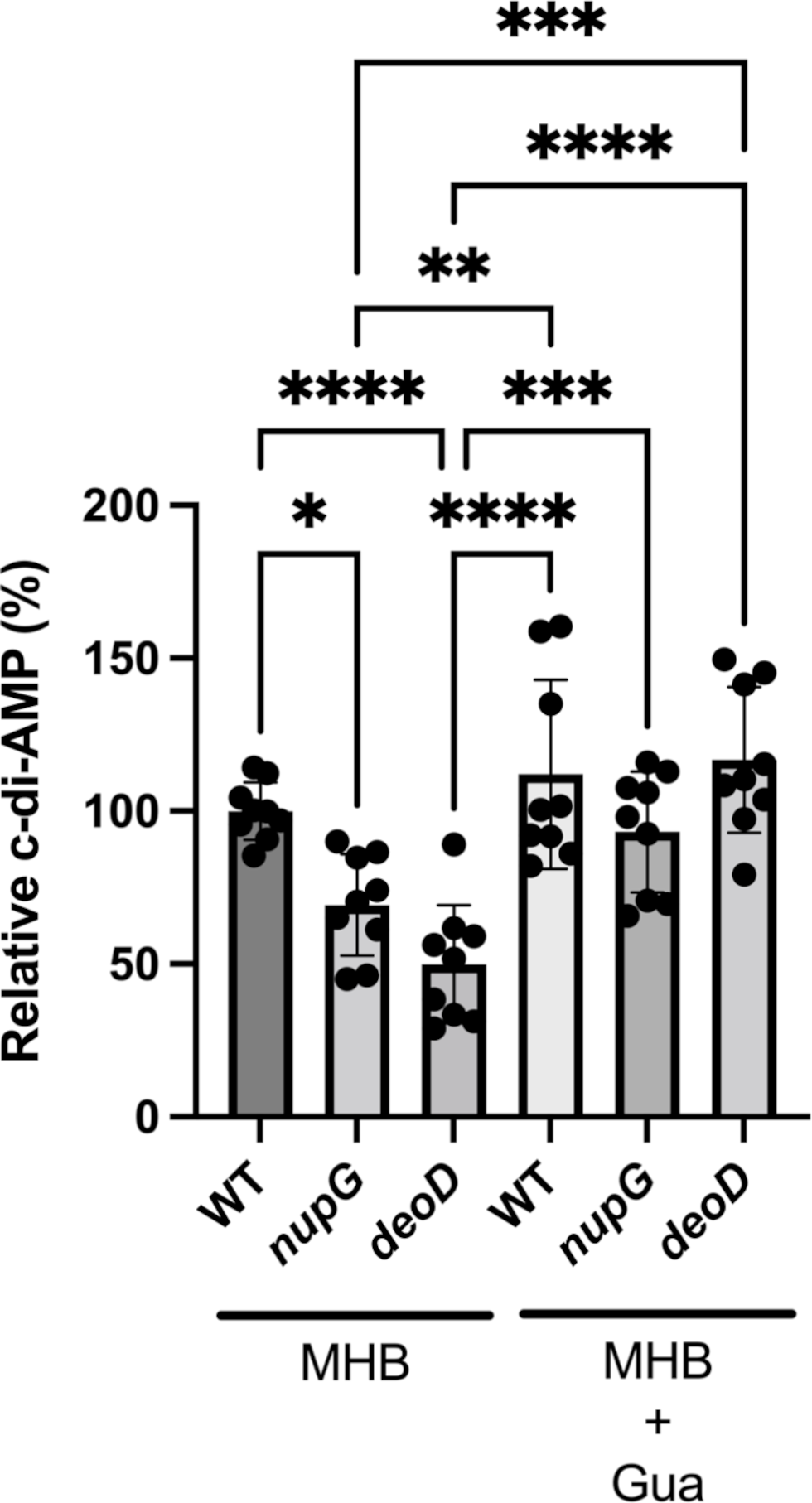

Supplement: FIG S7 [file mbio.02478-22-s0007.tif]

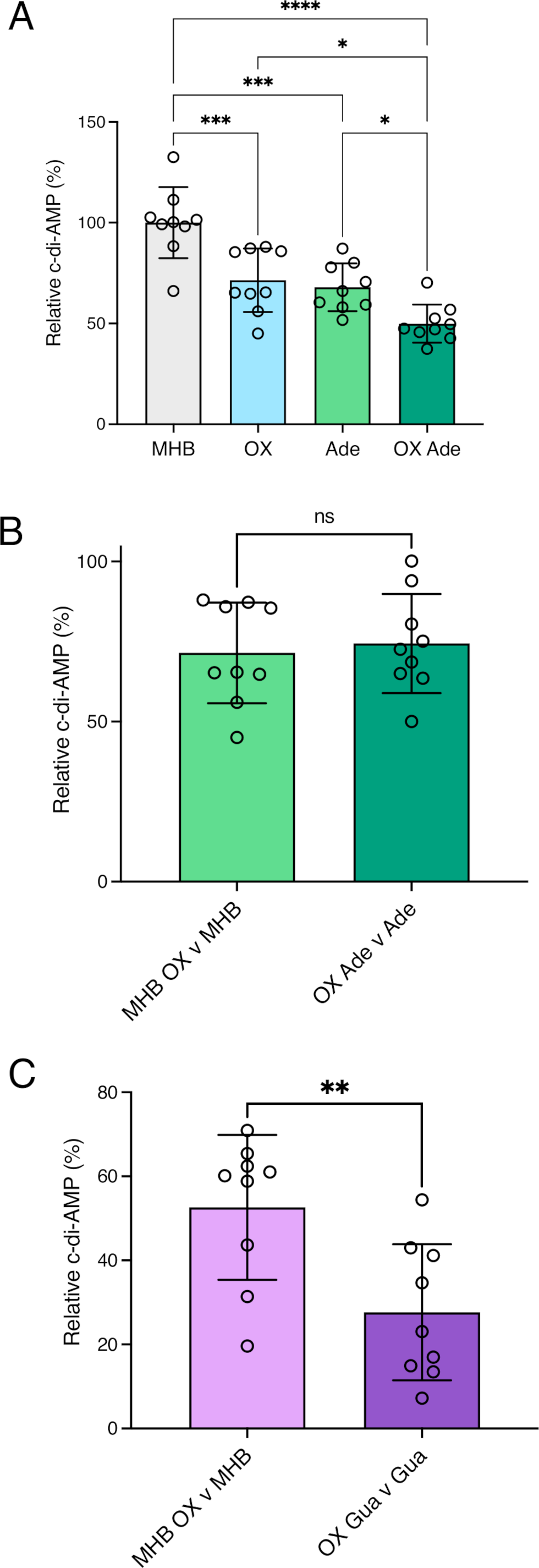

Supplement: FIG S8 [file mbio.02478-22-s0008.tif]

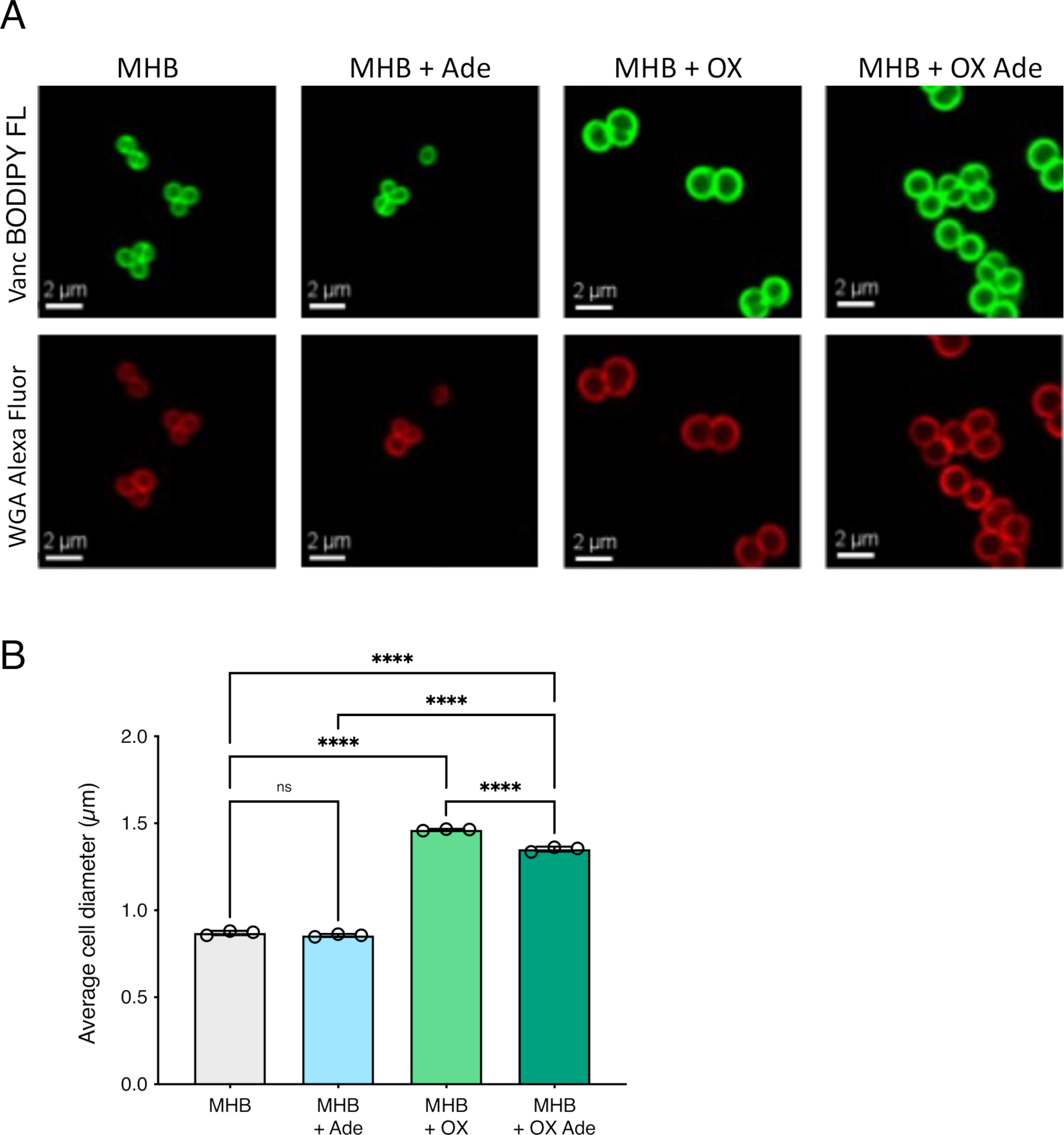

Supplement: FIG S5 [file mbio.02478-22-s0005.tif]
