# Supplementary material for: Purine Nucleosides Interfere with c-di-AMP Levels and Act as Adjuvants To Re-Sensitize MRSA To β-Lactam Antibiotics
Source: mBio. 2022 Dec 12;14(1):e02478-22. doi: 10.1128/mbio.02478-22 (PMC9973305; doi:10.1128/mbio.02478-22)
Supplement: TABLE S1 [file mbio.02478-22-s0009.docx]

**Table S1.** Bacterial strains and plasmids used in this study

| **Strains/plasmids** | **Relevant Details** |
| --- | --- |
| JE2 | USA300 cured of p01 & p03. Parent of Nebraska Transposon Mutant Library (NTML). |
| USA300 FPR3757 | Community associated MRSA isolate (5). SCC*mec* type IV. CC8. |
| MW2 | MRSA SCC*mec* type IV; CC1 (68) |
| DAR13 | MRSA reference isolate; SCC*mec* type IV; CC8 (69, 70) |
| DAR173 | MRSA reference isolate; SCC*mec* type IV; CC5 (69, 70) |
| DAR169 | MRSA reference strain; SCCmec type I; CC8 (69, 70) |
| DAR45 | MRSA reference isolate; SCC*mec* type II; CC30 (69, 70) |
| DAR22 | MRSA reference isolate; SCC*mec* type III; CC5 (69, 70) |
| *S. epidermidis* RP62A | ATCC 35984. Methicillin resistant, biofilm positive (71) |
| COL | MRSA reference strain; SCC*mec* type I; CC8 (72) |
| BH1CC | MRSA clinical isolate; SCC*mec* type II; CC8 (69) |
| 8325-4 | NCTC 8325 derivative cured of prophages (73), MSSA, CC8. |
| NE1868 *mecA* | JE2 *mecA*. Erm^r^. (44) |
| NE477 *deoD1* | JE2 *deoD1* (SAUSA300_0138). Erm^r^. (44) |
| NE529 *purF* | JE2 *purF*. Erm^r^. (44) |
| NE522 *purA* | JE2 *purA*. Erm^r^. (44) |
| NE950 *purB* | JE2 *purB*. Erm^r^. (44) |
| NE529 *purC* | JE2 *purC*. Erm^r^. (44) |
| NE581 *purD* | JE2 *purD*. Erm^r^. (44) |
| NE744 *purK* | JE2 *purK*. Erm^r^. (44) |
| NE1785 *purN* | JE2 *purN*. Erm^r^. (44) |
| NE1134 *purS* | JE2 *purS*. Erm^r^. (44) |
| NE353 *purH* | JE2 *purH*. Erm^r^. (44) |
| NE1101 *purM* | JE2 *purM*. Erm^r^. (44) |
| NE1464 *purL* | JE2 *purL*. Erm^r^. (44) |
| NE494 *purQ* | JE2 *purQ*. Erm^r^. (44) |
| NE1237 *purR* | JE2 *purR*. Erm^r^. (44) |
| NE1419 *nupG* | JE2 *nupG* (SAUSA300_0611). Erm^r^. (44) |
| NE283 *pbuG/stgP* | JE2 *pbuG/stgP* (SAUSA300_2207)*.* Erm^r^. (44) |
| NE280 *pbuX* | JE2 *pbuX* (SAUSA300_0387)*.* Erm^r^. (44) |
| NE650 *deoD2* | JE2 *deoD2* (SAUSA300_2091). Erm^r^. (44) |
| NE477 *deoD1* | JE2 *deoD1* (SAUSA300_0138). Erm^r^. (44) |
| USA300 R10 *hpt* S_71_C | USA300 6-thioguanine resistant mutant. Hpt S_71_C mutation (45) |
| USA300 R11 *hpt* T_61_P | USA300 6-thioguanine resistant mutant. Hpt T_61_P mutation (45) |
| NE1419 pLI50_*nupG* | NE1419 pLI50_*nupG* Erm^r^, Cm^r^ |
| NE650 pLI50_*deoD2* | NE650 pLI50_*deoD2*. Erm^r^, Cm^r^ |
| ANG3165 *rsh*_syn_ | USA300 LAC**rsh*_syn_. (25) |
| ANG1961 Δ*gdpP*::Km | USA300 LAC*Δ*gdpP*::*kan.* Kan^r^. (14) |
| ANG3664 *dacA* G_206_S | USA300 *dacA* G_206_S. (55) |
| *nupG*::Tn | *nupG*::Erm^r^ from NE1419 transduced into wild-type JE2 |
| *deoD2*::Tn | *deoD2*::Erm^r^ from NE1419 transduced into wild-type JE2 |
| MW2 *nupG*::Tn | *nupG*::Erm^r^ from NE1419 transduced into MW2 |
| MW2 *deoD2*::Tn | *deoD2*::Erm^r^ from NE650 transduced into MW2 |
| RN4220 | Restriction deficient derivative of *S. aureus* 8325 (73) |
| *E. coli* HST08 | TaKaRa *E. coli* HST08 Premium Electro-Cells |
| HST06 pLI50_*deoD2* | *E. coli* HST06 carrying pLI50_*deoD2*. Amp^r^ |
| HST06 pLI50_*nupG* | *E. coli* HST06 carrying pLI50_*nupG*. Amp^r^ |
| **Plasmids** |  |
| pLI50 | *E. coli* (Ap^r^)*-Staphylococcus* (Cm^r^)shuttle vector. |
| pLI50_*deoD2* | pLI50 carrying *deoD2* from JE2. Cm^r^. This study. |
| pLI50_*nupG* | pLI50 carrying *nupG* from JE2. Cm^r^. This study. |
