# Supplementary material for: Purine Nucleosides Interfere with c-di-AMP Levels and Act as Adjuvants To Re-Sensitize MRSA To β-Lactam Antibiotics
Source: mBio. 2022 Dec 12;14(1):e02478-22. doi: 10.1128/mbio.02478-22 (PMC9973305; doi:10.1128/mbio.02478-22)
Supplement: TABLE S2 [file mbio.02478-22-s0010.docx]

**Table S2.** Oligonucleotide primers used in this study.

| **Target gene** | **Primer name** | **Primer sequence (5’-3’)** |
| --- | --- | --- |
| *purA* | purA_Tn_check | TCATCAATCGTAGTAGTTGGGACACA |
| *purB* | purB_Tn_check | GGAGAAGTAAAGTGAATAAATTCTGGTA |
| *purC* | purC_Tn_check | CCACTAGCAATATTACGAACAACTACTTC |
| *purD* | purD_Tn_check | GTCATTAGCGAGAACTCTTCACC |
| *purF* | purF_Tn_check | CGAAGAATGTGGCGTGTTTGG |
| *purH* | purH_Tn_check | CGCTTCATCATATTCTGCAGTATGC |
| *purK* | purK_Tn_check | CTGAAGATTGTCCATGTAGATACGTTGC |
| *purL* | purL_Tn_check | CGTACTACCGATGCTTGAAGTCCT |
| *purM* | purM_Tn_check | GCAAATCCAGCTACATCATATTCGC |
| *purQ* | purQ_Tn_check | CCAGGTTCGAATTGTGATAGAGACA |
| *purS* | purS_Tn_check | GGATGAAATATCTCCAGATACATGTCG |
| *purN* | purN_Tn_check | CACGCGCTTTGAAGATTTTAGCAGAAC |
| *deoD1* | deoD1_Tn_check | GAAGCAATTGGCGCAAAATGAC |
| *deoD2* | deoD2_Tn_check | CACCACATATTAACCAAATGGAG |
| *nupG*  *pbuX*  *pbuG*  *relA* | nupG_Tn_check  pbuX_Tn_check  pbuG_Tn_check  relA_Tn_check | GAGCAGCCAATGGTATCGCT  GGGAGAAGAATAATGAAAAATTTAATCC  CTGCACTTGTTACAACAGCGATTAACGG  GCGAGTGATGTTATTGAGTTGCC |
| **Infusion cloning primers** | |  |
| *deoD2* | INF_deoD2_F | TCGTCTTCAAGAATTACATGCGTTGGATTCACACA |
|  | INF_deoD2_R | TACCGAGCTCGAATTTGGTAATGCTGGCGATGATG |
| *nupG* | INF_nupG_F | TCGTCTTCAAGAATTGGTAACAGGTAAAGGTACGCA |
|  | INF_nupG_R | TACCGAGCTCGAATTATAGACGACATGCCTGGACA |
